# Can knowledge based treatment planning of VMAT for post-mastectomy locoregional radiotherapy involving internal mammary chain and supraclavicular fossa improve performance efficiency?

**DOI:** 10.3389/fonc.2023.991952

**Published:** 2023-04-03

**Authors:** Reena Phurailatpam, Muktar kumar Sah, Tabassum Wadasadawala, Asfiya Khan, Jithin Palottukandy, Umesh Gayake, Jeevanshu Jain, Rajiv Sarin, Rima Pathak, Revathy Krishnamurthy, Kishore Joshi, Jamema Swamidas

**Affiliations:** ^1^ Department of Radiation Oncology, Advanced Centre for Treatment, Research and Education in Cancer (ACTREC), Tata Memorial Centre, Homi Bhabha National Institute, Mumbai, India; ^2^ Department of Radiation Oncology, Tata Memorial Centre, Homi Bhabha National Institute, Mumbai, India

**Keywords:** knowledge-based planning, chest wall, internal mammary nodal (IMN) region and supra-clavicular fossa (SCF), validation, left-side, post-mastectomy radiotherapy

## Abstract

**Introduction:**

To validate and evaluate the performance of knowledge-based treatment planning for Volumetric Modulated Arc Radiotherapy for post-mastectomy loco-regional radiotherapy.

**Material and methods:**

Two knowledge-based planning (KBP) models for different dose prescriptions were built using the Eclipse RapidPlanTM v 16.1 (Varian Medical Systems, Palo Alto, USA) utilising the plans of previously treated patients with left-sided breast cancer who had undergone irradiation of the left chest wall, internal mammary nodal (IMN) region and supra-clavicular fossa (SCF). Plans of 60 and 73 patients were used to generate the KBP models for the prescriptions of 40 Gy in 15 fractions and 26 Gy in 5 fractions, respectively. A blinded review of all the clinical plans (CLI) and KBPs was done by two experienced radiation oncology consultants. Statistical analysis of the two groups was also done using the standard two-tailed paired t-test or Wilcoxon signed rank test, and p<0.05 was considered significant.

**Results:**

A total of 20 metrics were compared. The KBPs were found to be either better (6/20) or comparable (10/20) to the CLIs for both the regimens. Dose to heart, contralateral breast,contralateral lung were either better or comparable in the KBP plans except of ipsilateral lung. Mean dose (Gy) for the ipsilateral lung are significantly (p˂0.001) higher in KBP though the values were acceptable clinically. Plans were of similar quality as per the result of the blinded review which was conducted by slice-by-slice evaluation of dose distribution for target coverage, overdose volume and dose to the OARs. However, it was also observed that treatment times in terms of monitoring units (MUs) and complexity indices are more in CLIs as compared with KBPs (p<0.001).

**Discussion:**

KBP models for left-sided post-mastectomy loco-regional radiotherapy were developed and validated for clinical use. These models improved the efficiency of treatment delivery as well as work flow for VMAT planning involving both moderately hypo fractionated and ultra-hypo fractionated radiotherapy regimens.

## Introduction

Breast cancer is the most commonly diagnosed cancer in women (approximately 2 million cases worldwide in 2020) and it accounts for 6.9% of the total cancer-related deaths (1).

Breast cancer screening leading to early diagnosis and effective treatment strategies have led to an improvement in the prognosis and survival rates, especially in the western world. However, in low and middle income countries (LMICs), majority of women still have to undergo mastectomy as they present with a locally advanced stage, often with internal mammary nodal (IMN) involvement, which is picked up on cross-sectional imaging done for disease staging. The loco-regional radiotherapy (LRRT) in such cases encompasses Post-Mastectomy Radiation Therapy (PMRT), including the regional nodes. As generally, the axilla is addressed surgically, regional nodal irradiation (RNI) often includes targeting the internal mammary node(IMN) and the supraclavicular fossa (SCF). Internal Mammary Nodal (IMN) Irradiation, particularly in patients with left-sided primary disease, is one of the most challenging scenarios in adjuvant radiation therapy owing to the close proximity of the target to the critical organs at risk, namely the heart, left anterior descending artery (LADA), lungs, and contralateral breast.

Traditionally, 3-dimensional conformal radiotherapy (3DCRT) delivered using partially wide tangents was one of the most common techniques employed for treating the chest wall along with the internal mammary region. This technique restricts the dose to the OARs (organs at risk), especially the heart and LAD, but compromises the target coverage and dose homogeneity (2). The combination of photon beam and electron beam has also been used for IMNI but with the limitations of over or under dosing at the photon-electron junction along with a high dose to the anterior myocardium. Rotational intensity modulated radiotherapy (IMRT) provides a viable solution in such a scenario by providing better target coverage, improved dose conformity, as well as homogeneity and better sparing of the OARs (3, 4). The absence of any form of junction and ease of setup are the added advantages. Volumetric modulated arc therapy (VMAT) is a commonly used technique to deliver IMRT.

A typical VMAT plan optimization requires multiple iterations, which makes it a time-consuming process. Variation in patient anatomy, skills and experience of the planner, clinical goals, and dose constraints are some of the parameters that affect the plan quality and make treatment planning laborious. Nelms et al. have stated that inter-planner variation, even within the same institute, is very evident in planning as each planner approaches plan optimization in a different manner using different plan optimization parameters, objectives, and priorities (5). David et al. have also stated that the dependence of the radiotherapy planning process on the planner’s experience has been increasing (6). Li et al. have also reported significant inconsistencies in plan quality and dose in the normal brain among VMAT brain stereotactic plans generated manually by three different institutions (7). They have, however, stated that automated planning has improved out-of-target dose and has the potential to help standardise the quality of care for patients receiving VMAT-based multi-target SRS. Knowledge-based planning has, thus, evolved as a way to efficiently create plans of uniform quality by reducing the inter-planner variability and the duration of the optimisation process. Scaggion et al. also reported that KBP can be used as a valuable tool to leverage the planning skills of less experienced planners, thereby ensuring better uniformity of treatment plan quality (8).

KBP models have been reported in the literature for multiple sites like head and neck cancer, prostate cancer, gynaecological cancer, and even breast cancer. Knowledge-based planning (KBP) models are reported in the literature for whole breast irradiation along with draining lymph nodes, bilateral breast radiotherapy, and chest wall irradiation without IMN targets. This study aims at evaluating the knowledge-based planning model, created using a commercial KBP tool RapidPlan™ (RP) provided with the Eclipse treatment planning system (Varian Medical Systems, Palo Alto, USA), for postmastectomy loco-regional radiation therapy including internal mammary nodes and supraclavicular nodes, and thereby validating it for clinical implementation. The complexity metrics of KBP plans are compared with clinical plans (9, 10). Patient specific quality assurance of KBP as well as clinical plans (CLI) is done using Arc Check phantom (Sun Nuclear Corporation, USA) and SNC patient software. To the best of our knowledge, such an investigation on dosimetric validation and deliverability check of the KBP plans for 26 Gy/5 fractions in comparison to CLI plans has not been reported though there are many papers on the conventional fractionation for 40 Gy/15 fractions.

## Materials and methods

This retrospective dosimetric study is a part of the KBP project that was approved by the Institutional Ethics Committee for which consent waiver was granted. Two knowledge-based planning (KBP) models for different dose prescriptions (40 Gy in 15 fractions and 26 Gy in 5 fractions) were built using RapidPlan™ v16.1 Eclipse Treatment Planning System (Varian Medical Systems, Palo Alto, USA) based upon previously treated patients with left-sided breast primary who had undergone irradiation of the left chest wall, internal mammary nodal region and supra-clavicular fossa. All consecutive patients with left sided breast cancer treated with VMAT at our institute from 2018 to 2021 were used for making KBP models and patients treated from 2020 to 2022 were screened for the validation. Patients in whom the target volumes encompassed the left chest wall, SCF and IMN and were treated with free breathing were considered as cases for the study. Other patients, including additional targets or not irradiating IMN were excluded.

As breast cancer patients with locally advanced disease routinely undergo axillary dissection, axillary radiotherapy is avoided to minimise the risk of lymphedema. RapidPlan™ is a commercially available module with Eclipse treatment planning system which models the data from previous patients and gives the DVH estimation of the volumes of interest prior to planning based on the various geometric and dosimetric parameters extracted from the input treatment plans. The patients incorporated for KBP model training were planned using Volumetric Modulated Arc Therapy (VMAT, Photon Optimizer, Acuros-XB, Eclipse v 16.1, Varian Medical Systems, Palo Alto, USA). The standard fractionation for IMNI is 40 Gy in 15 fractions at our institute. However, during the COVID-19 pandemic, the fast forward fractionation was adopted for breast radiotherapy based on the modified UK recommendations released during the pandemic in April 2020 (11). Hence the patients with locally advanced breast cancer requiring IMNI were also treated with fast forward fractionation during the pandemic.

### Contouring

Clinical Target volumes (CTV) for Left chest wall (CTV_CW), and SCF (CTV_SCF) were delineated according to the ESTRO guidelines (12). For the internal mammary nodal targets, CTV comprised of the IMN vessels (CTV_IMN) starting from the caudal limit of CTV_SCF to cover the upper three intercostal spaces (up to the fourth rib) as the most common location of the IMN was in the first intercostal space. A margin of 5 mm was given to the CTV to delineate all the PTVs. PTVs were cropped 3mm from the skin. The organs at risk that were delineated included left anterior descending artery (LAD), heart, left lung, right lung, contralateral breast (right breast), thyroid, oesophagus and spinal cord.

### KBP model: training, outlier analysis and model objectives generation

The two KBP models were trained using the plans of treated patients which were made following a consistent planning protocol. Flattened 6 MV beam was used to plan the cases with 2 to 4 partial arcs of arc length between 180˚ to 220˚with the isocentre placed at the centre of PTV chest wall. The collimator angle was varied between 5˚ to 15˚.A total of 60and 73 patients were selected for generating the KBP modelsfor40 Gy in 15 fractions and 26 Gy in 5 fractions model respectively. The inclusion criteria for plans to be used in the training set were based on their compliance with the institutional dose constraints. (**Table 1**)

**Table 1:** Clinical Goals for the targets and Dose Constraints for the Organs at risk.

TARGET	Clinical Goals (For 26 Gy/5 # and 40 Gy/15#)	
	Desirable	Acceptable
PTV LT CW	V95% ≥ 95%	V95% ≥ 92%
PTV LT SCF	V95% ≥ 95%	V95% ≥ 92%
PTV LT IMN	V95% ≥ 95%	V95% ≥ 90%
Organs at risk	Dose Constraints	
	26Gy/5#	40Gy/15#
LAD	Dmax≤ 13 Gy ± 2 Gy	Dmax≤ 18 Gy ± 4 Gy
Heart	Dmean≤ 2.5 Gy ± 0.5 Gy	Dmean≤ 5 Gy ± 1 Gy
Contralateral Breast (Right)	Dmean≤ 2 Gy ± 0.5 Gy	Dmean≤ 3 Gy ± 0.5 Gy
Contralateral Lung (Right)	Dmean≤ 2 Gy ± 1 Gy	Dmean≤ 4 Gy ± 1 Gy
Ipsilateral Lung (Left)	Dmean≤ 7 Gy ± 1 Gy V3Gy≤ 55% ± 10% V6Gy≤ 35% ± 10%	Dmean≤ 10 Gy ± 2 Gy V5Gy ≤ 35% ± 10% V18Gy≤ 30% ± 5%
Oesophagus	Dmax≤ 26 Gy ± 1 Gy	Dmax≤ 40 Gy ± 1 Gy
Thyroid	Dmax≤ 26 Gy ± 1 Gy	Dmax≤ 40 Gy ± 1 Gy
Spinal Cord	Dmax≤ 10 Gy ± 3 Gy	Dmax≤ 18 Gy ± 4 Gy
Body	Dmax≤ 107% ± 5% V107% ≤ 2cc ± 3cc	Dmax≤ 107% ± 5% V107% ≤ 2cc ± 3cc

A detailed explanation of the configuration and training process of KBP models has been given by various authors (13, 14). RapidPlan Model creation is an iterative process that includes training, outlier analysis and re-training based on the outlier statistics. The RP models created were analysed for the outliers using the regression and residual plots of the OARs. Regression models created by KBP between geometric and dosimetric components can detect outliers and thereby improve the capability of KBP. The geometric outliers were retained in the training set, but the dosimetric outliers were identified for further modification. The plans of the dosimetric outliers were studied for root-cause analysis and if required, the cases were re-planned and put into the training set again.The coefficients of determination (R^2^), Chi-square (χ^2^), and the mean square error (MSE)are the in-built statistical tools provided with RP module.For the target volumes, suitable objective priorities established from clinical experience were used while for the OARs, optimization constraints such as ‘line objectives’, mean dose and upper dose constraints were used in accordance with the clinical goals. The priority values were automatically generated. Upper dose objectives were placed in addition to the line objective along the inferior DVH prediction boundary for OARs. Normal tissue objective settings were based on clinical experience so that the dose spill outside the target volumes was controlled. The priority was set similar to that of the PTVs while the distance from the target border was set as 0.5 cm with a start dose of 100%, end dose of 60% and a fall off criteria of 0.5.

### Validation

Thirteen patients were used for the validation of the 26 Gy/5 fractions KBP model, while ten patients were used to validate the 40 Gy/15 fractions model. These patients were totally independent of the training set and were planned manually as well as using the respective KBP models. The plan parameters, like the energy and the arc geometry, were kept exactly the same in the manual as well as in KBP plans. KBP plans were generated in a single optimization run without any manual intervention.

The CLI plans and the KBP plans were compared on the basis of various dosimetric parameters like the conformation number, homogeneity index, total MUs(monitor units), and various dose levels for OARs. Conformation number is considered as it takes into account irradiation of both target volume and healthy tissues. To check the statistical significance of the difference between CLI and KBP plans, either a two-tailed paired t-test or a Wilcoxon signed rank test was done based on the normality of the data distribution and the differences were reported with a 95% confidence interval. (**Table 2**). For the qualitative comparison of KBP with CLI, a visual check of axial dose distribution was done. The definitions of the various indices used for the data analysis have been reported below:

**Table 2:** Mean ± standard deviation of the dose-volume parameter of clinical plans as compared to validation plans. p values are given for Clinical plan vs Predicted plan (p<0.05 considered as significant).

Organ & Dose Parameter	Clinical Plan	26 Gy/5 Fractions Predicted Plan	p value	Clinical Plan	40 Gy/5 Fractions Predicted Plan	p value
PTV LT CW V95% (%)	93.24 ± 2.41	93.2 ± 3.05	0.947	96.59 ± 1.62	95.25 ± 2.21	0.053
PTV LT SCF V95%(%)	94.95 ± 1.43	97.27 ± 1.68	0.000	98.03 ± 1.30	97.63 ± 1.72	0.301
PTV LT IMN V95%(%)	89.48 ± 2.94	91.21 ± 2.49	0.171	92.76 ± 2.12	92.57 ± 2.45	0.819
LAD Dmax(Gy	15.1 ± 3.07	13.16 ± 2.43	0.001	17.19 ± 2.70	16.27 ± 2.30	0.144
Heart Dmean(Gy)	2.92 ± 0.54	2.79 ± 0.55	0.060	3.45 ± 0.69	3.37 ± 0.73	0.426
Contralateral Breast (Right) Dmean(Gy)	2.14 ± 0.34	2.27 ± 0.24	0.162	3.14 ± 0.23	2.85 ± 0.25	<0.000
Contralateral Lung (Right)Dmean(Gy)	2.377 ± 0.39	2.162 ± 0.23	0.012	3.46 ± 1.04	3.06 ± 1.04	0.039
IpsilateralLung : Dmean(Gy) V3Gy/V5Gy (%) V6Gy/V18Gy (%)	6.67 ± 0.71 53.68 ± 3.68 35.88 ± 3.72	7.48 ± 0.39 61.49 ± 2.78 41.54 ± 2.39	<0.001 <0.001 <0.001	9.10 ± 0.85 48.04 ± 3.55 17.39 ± 2.64	9.71 ± 0.70 51.60 ± 4.45 19.29 ± 2.58	0.042 0.078 0.022
Oesophagus: Dmax(Gy)	25.65 ± 1.53	25.95 ± 1.64	0.063	39.89 ± 3.77	39.54 ± 4.24	0.173
Thyroid: Dmax(Gy)	26.35 ± 0.57	26.93± 0.21	0.005	41.09 ± 0.46	41.03 ± 0.47	0.697
Spinal cord: Dmax(Gy)	11.81± 1.79	10.97 ± 2.07	0.043	17.32 ± 3.15	13.54 ± 1.57	0.002
Body V107% (cc)	0.4 ± 0.733	0.489 ± 1.33	0.965	0.21 ± 0.35	0.53 ± 0.89	0.236
Homogeneity Index	0.121 ± 0.020	0.118 ± 0.023	0.390	0.085 ± 0.013	0.0931 ± 0.016	0.022
Conformation Number	0.931 ± 0.022	0.934 ± 0.028	0.470	0.878 ± 0.019	0.864 ± 0.018	0.009
Complexity metrices	0.14 ± 0.02	0.12 ± 0.01	0.014	0.13 ± 0.12	0.12± 0’01	0.049
Total MUs	1994.461 ± 275.249	1704.538 ± 315.274	0.013	948.1 ± 141.506	897.8 ± 149.924	0.010
Gamma	96.21( ± 4.85)	96.87( ± 1.77)	0.598	97.92 ± 1.39	98.36 ± 1.19	0.031

### Conformation number, CN = (VT_ref_/VT) x (VT_ref_/V_ref_)

where VT_ref_ is the volume of target receiving a dose equal to or greater than the reference dose, VTis the total target volume, V_ref_ is the volume receiving a dose equal to or greater than the reference dose. Total Target volume (VT) is the sum of PTV_CW, PTV_SCF and PTV_IMN.

### Homogeneity index, HI = (D_2_ -D_98_)/Dp ×100

where D _2_ = minimum dose to 2% of the target volume indicating the “maximum dose”,

D _98_ = minimum dose to the 98% of the target volume, indicating the “minimum dose” and

D _p_ = prescribed dose.

The Ideal value for Conformation number (CN) is 1 and it ranges are from 0 to 1.Ideal value of Homogeneity index (HI) is zero.

### Computation of plan complexity and the delivery quality assurance

The plan’s complexity was checked by total MUs and aperture complexity metrics. The aperture complexity metric is the MU weighted sum over all control points of the perimeter by area for the aperture. The aperture complexity parameter was calculated by


$$M=\frac{1}{MU}\sum_{i=1}^{N}MUi\times \frac{yi}{Ai}$$

Where y_i_ is the perimeter and A_i_ is the area of the aperture at the control point. MU represents the total MU for the plan while MU_i_ is MU delivered at the i^th^ control point. The plan complexity is calculated using the Eclipse Application Programming Interface (Eclipse ESAPI) version 16.1 [9, 10].

The plan deliverability was checked by performing patient specific QA and calculating the gamma passing rate (GPR) using the Arc Check phantom (Sun Nuclear Corporation, USA) and SNC patient software.

### Assessment of the impact of KBP implementation on clinical work flow

The impact of KBP on the clinical work flow in terms of plan uniformity and the time taken for generating a clinically acceptable plan in view of the experience of the planners was also evaluated.

### Blinded review of plans

To avoid inclination towards any specific planning technique, blinded review of the CLI and KBP plans for both the models was done by experienced clinicians. The CLI and KBP plans were randomly renamed as A or B, and the clinicians were asked to select the better plan. It was slice by slice evaluation of dose distribution for coverage and overdose volume as well as dose to OARs.

## Results

**Figure 1:**
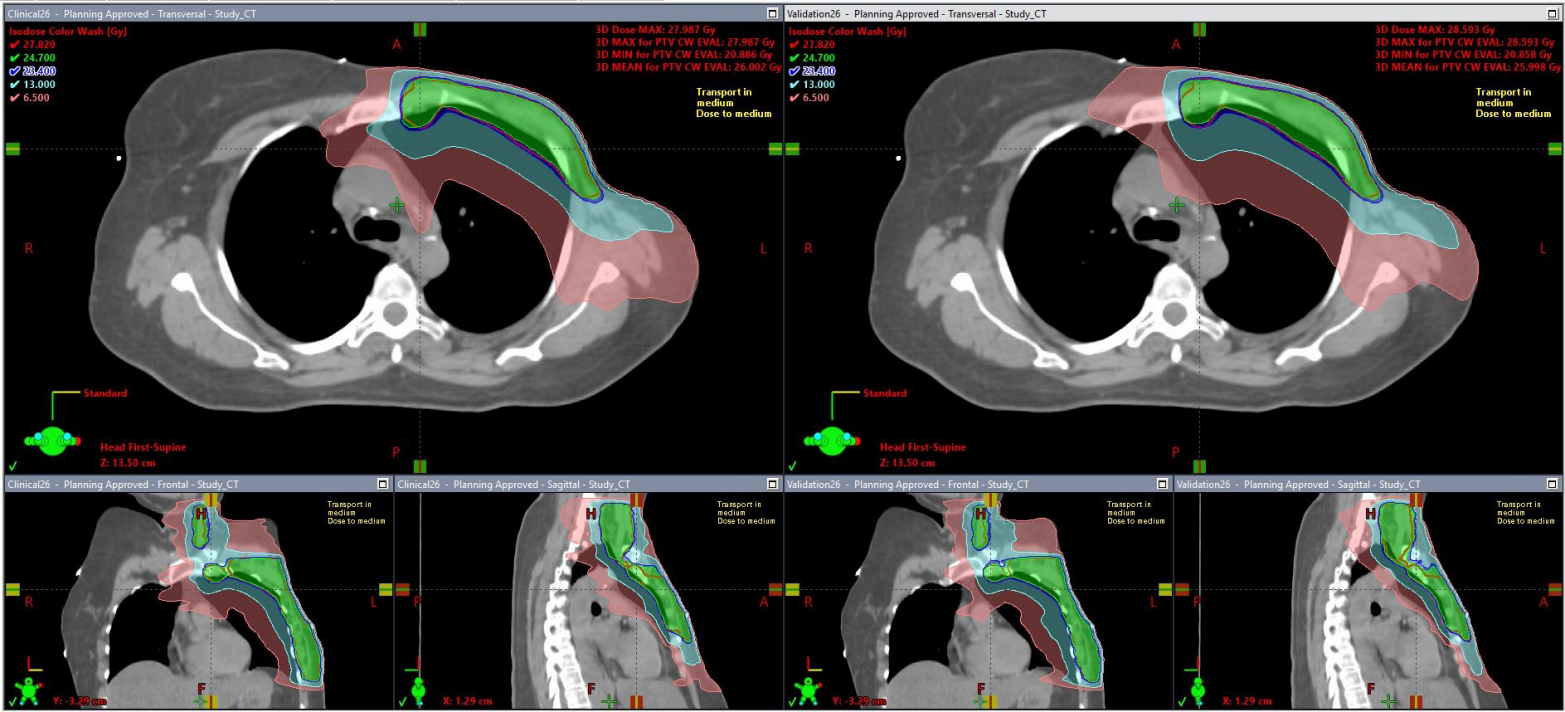
Representative dose distribution for comparison of CLI vs KBP_26Gy in 5 fractions.

**Figure 2:**
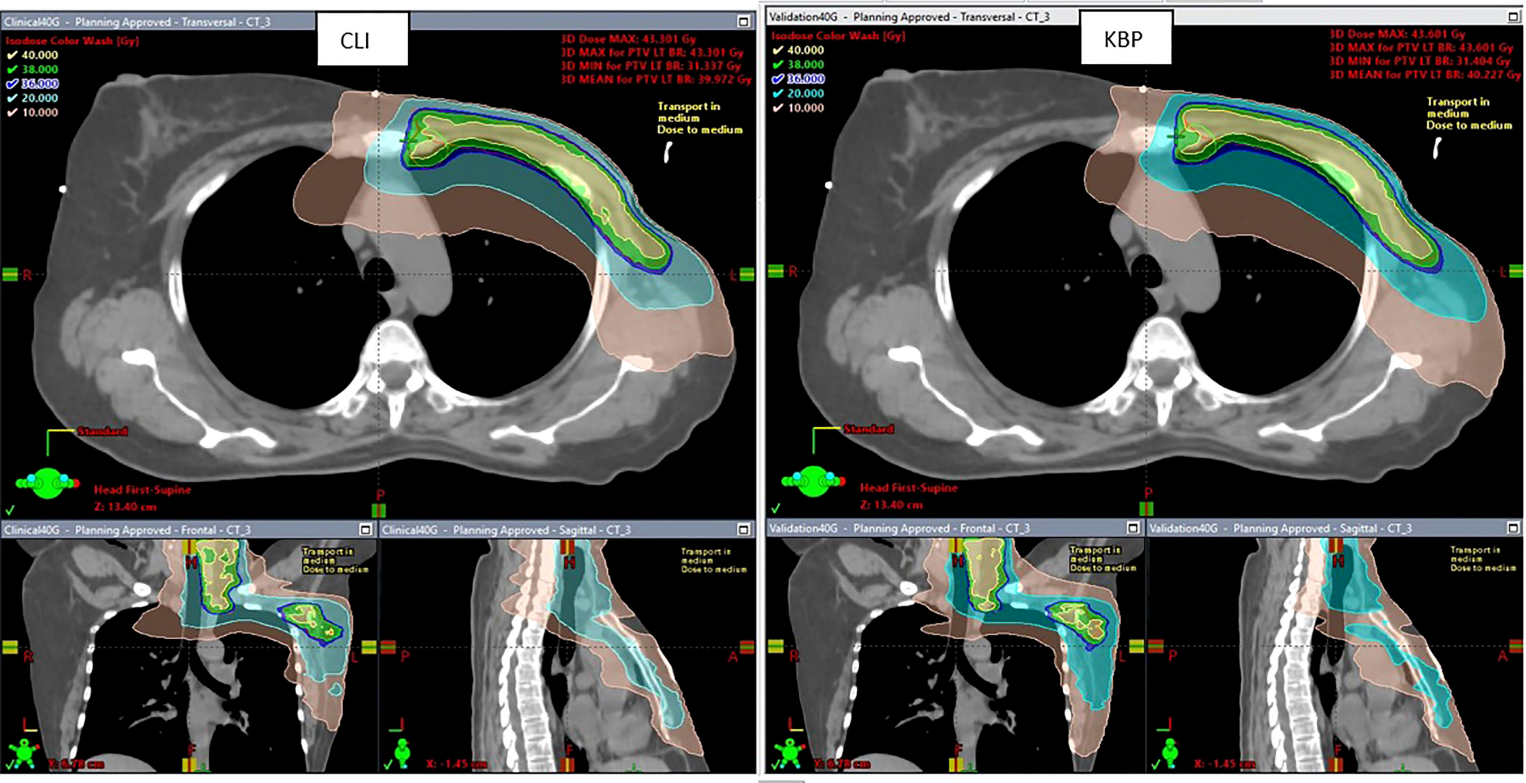
Representative dose distribution for comparison of CLI vs KBP_40 Gy in15 fractions.

**Figure 3:**
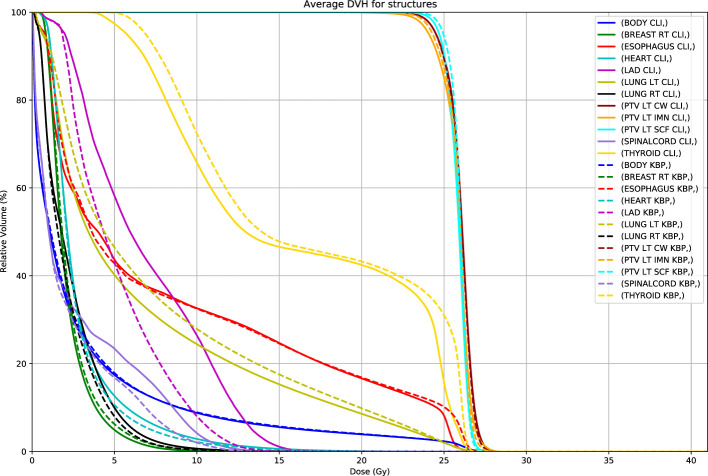
Average DVH comparison of CLI and KBP_26Gy in 5 fractions.

**Figure 4:**
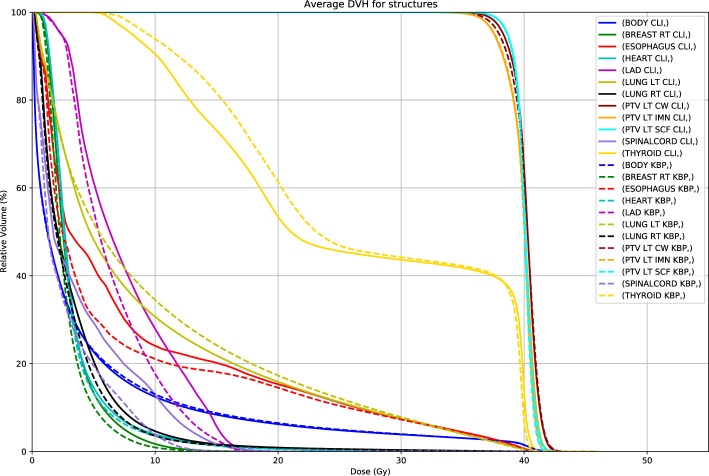
Average DVH comparison of CLI and KBP_40Gy in 15 fractions.

The CLI as well as the KBP plans could achieve all the clinical goals, and a representative dose distribution for their comparison is shown in **Figures 1**, **2** for 26Gy/5fractions and 40 Gy/15fractions, respectively. The mean DVH plots for each target volume and OARs are also shown in **Figures 3**, **4** for the 26Gy/5 fractions and 40Gy/15 fractions, respectively.

### Model 1 (KBP model for 26Gy in 5 fractions)

In terms of the target coverage and dosimetric indices, clinical goals are achieved by the CLI as well as the KBP plans. KBP plans give comparable coverage to the targets when compared to CLI.Coverage of Targets (PTV left CW and PTV left IMN) coverage are not significantly different between CLI and KBPs while coverage of PTV SCF (%) is significantly better in KBP (97.27 ± 1.68) as compared to CLI (94.95 ± 1.43) with p=0.000.At the same time HI and CN are comparable. Mean Heart dose (Gy) is not significantly lesser in KBP (2.79 ± 0.55) as compared to CLI (2.92 ± 0.54) with p=0.060.Whereas the dose parameters for the contralateral lung(p=0.012), LAD(p=0.001), and spinal cord(p=0.043) were significantly lower in KBP than in CLI. However, mean dose (Gy) for the ipsilateral lung are significantly higher in KBP (7.48± 0.39) than in CLI (6.67 ± 0.71) with p<0.001.Contralateral breast (p=0.162) and oesophagus (p=0.063) dose are comparable.Thyroid dose is significantly more in CLI (p=0.005).CLI plans are found to be more complex than KBPs as MUs (p=0.013) and complexity indices (p=0.014) are significantly higher. The GPR for delivery quality assurance (DQA) were comparable (p=0.598).

### Model 2 (KBP model for 40 Gy in 15fractions)

KBP plans give comparable coverage to the targets when compared to CLI. For heart, LAD, thyroid and oesophagus, doses are comparable in KBP and CLI. However, the dose parameters for contralateral breast (p<000), contralateral lung (p=0.039) and spinal cord (p=0.002) are significantly less in KBP plans than in CLIs thereby favoring KBP plans. For Ipsilateral lung, mean dose (Gy)in KBP plan(9.71 ± 0.70) significantly higher than in CLI (9.10 ± 0.85)with p=0.042.However volume of ipsilateral lung getting 5Gy dose (V5) is comparable with p=0.078. Similar to the observation in model 1, CLI plans are found to be more complex than the KBP plans as MUs(p=0.01) and complexity indices(p=0.049) are significantly higher for CLI as compared to KBPs. GPR for DQA are significantly better for KBPs(p=0.031). CN (p=0.009) and HI (p=0.022) values significantly favored CLI plans.

### Impact of KBP on clinical work flow

The plans used for training both the KBP models have been planned by highly experienced planners (at least 10 years’ experience). The time taken for creating a manual plan is calculated to be approximately 4-5 hrs. During the manual planning, planners have to create many optimization structures to control the dose spill outside the PTVs and to create dose gradients in the OARs. To achieve the clinical goals, the optimizer has to constantly change constraints and penalties to target volumes and OARs using the hit-and-trial method. KBP plans have been created by inexperienced planners. The optimization is done in a single run, so a clinically acceptable plan is created in 20–30 minutes. It is thus observed that the time required to achieve clinically acceptable plans has been drastically reduced.

### Blinded review

**Figure 5:**
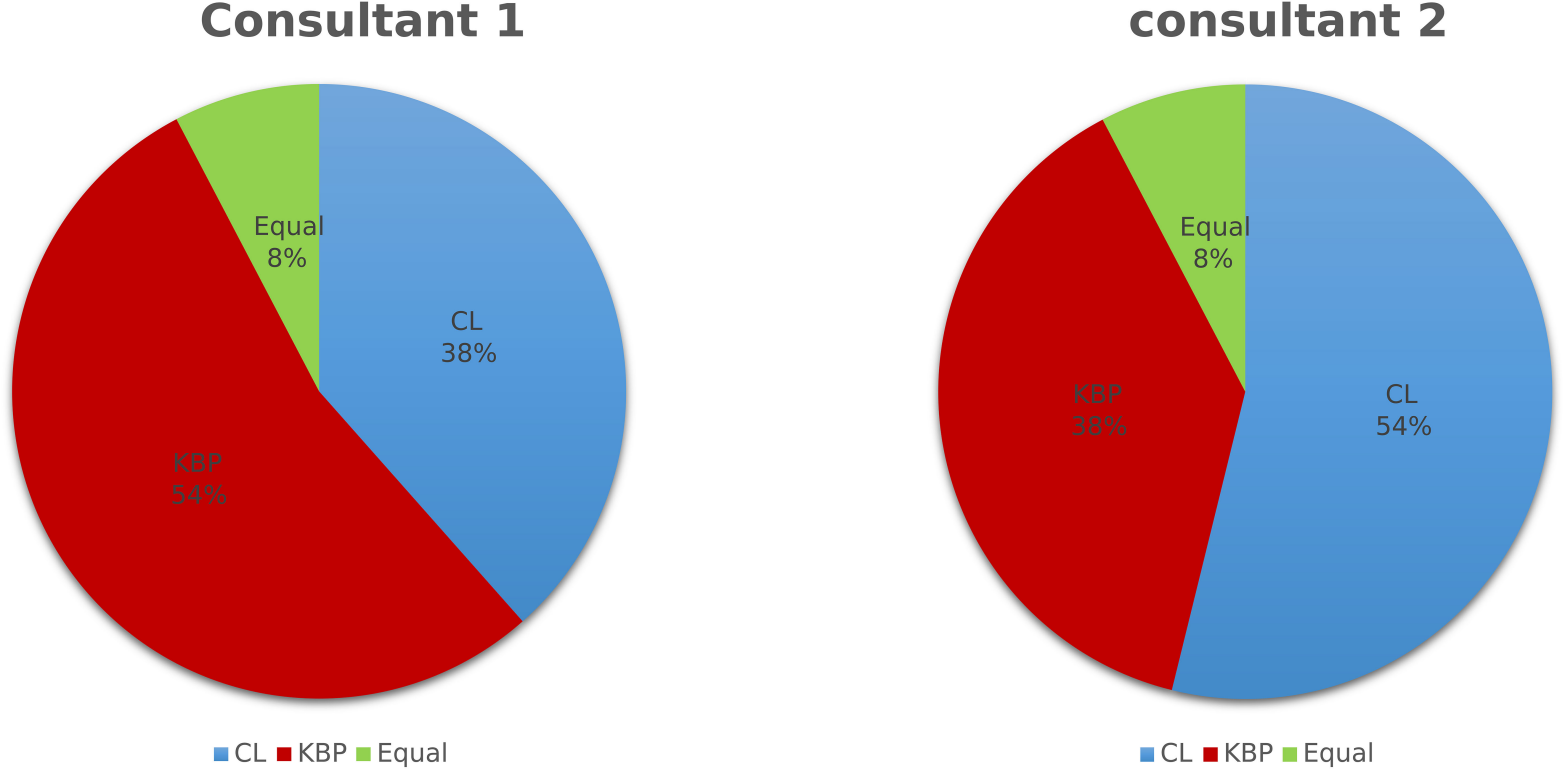
Blinded review result for 26Gy in 5 fractions.

For Model 1 (26Gy in 5 fractions), out of the 13 validation cases, consultant 1 found 7 CLI plans and 5 KBP plans better on comparison, while for 1 validation case, both the plans were of equal quality. Consultant 2 chose 5 CLI plans and 7 KBP plans, and in one case, both plans were of equal quality. The result is depicted in **Figure 5**.

**Figure 6:**
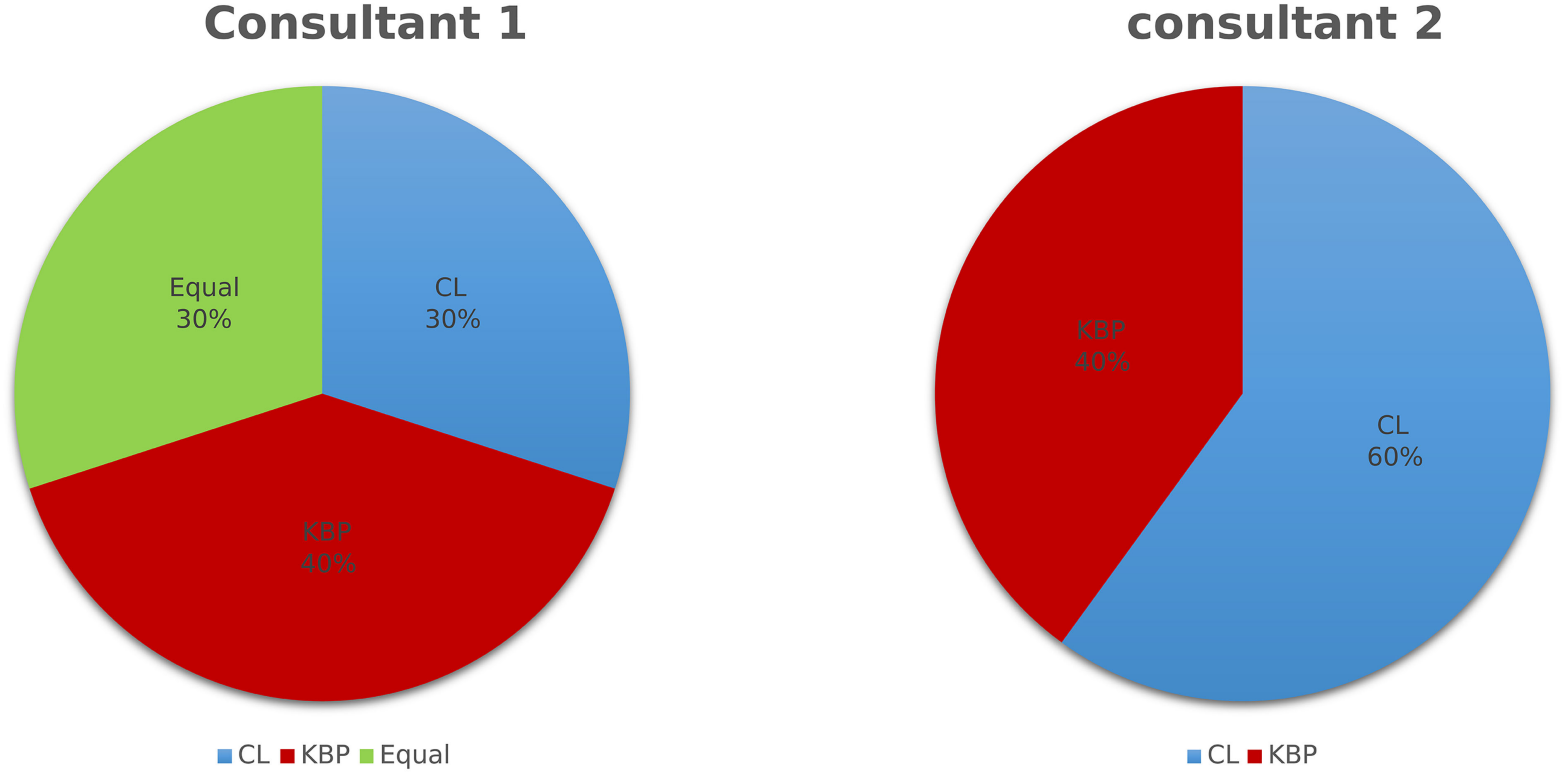
Blinded review result for 40 Gy in 5 fractions.

For Model 2 (40Gy in 15 fractions), out of the 10 validation cases, 3 CLI plans and 4 KBP plans were selected, while 3 plans were found to be of equivalent quality by consultant 1. Consultant 2, on the other hand, preferred 6 CLI plans and 4 KBP plans. The result is depicted in **Figure 6**.

## Discussion

Keeping pace with the numerous advancements in the field of radiation oncology, artificial intelligence and machine learning have proven to have great potential for impacting the patient workflow in cancer care, especially for busy radiation oncology setups providing services to treat the most prevalent disease sites like head and neck, breast, gynecological, or prostate cancers. There have been various published reports on the automated planning models for breast cancer, as summarized in **Table 3** (14–21). Out of 8 papers discussed, 6 papers are on RapidPlan™ while 2 papers are on Raystaion TPS and MD Anderson Cancer Center Auto Plan (MDAP) system. Many authors have hypothesized the potential of automated radiotherapy treatment planning for increasing consistency, improving plan quality, and reducing workloads for all routinely challenging treatments involving complex anatomical sites or involving multiple dose levels (15, 19). Most of these papers reported on the conventional fractionation regime while we are reporting on the KBPs for both moderately hypo fractionated and ultra-hypo fractionated (fast forward) regimens. The model for fast forward fractionation (26 Gy in 5 fractions) has not been reported in literature. Anatomical differences amongst patients are taken into account and extrapolated in the evaluation of DVH for the treating patients. Our study is not restricted to the dosimetric validation but also includes checks on the deliverability of plans in terms of plan complexity, MU, and gamma passing rate. Patients undergoing mastectomy and presenting with N3 disease in view of an initial positive IMN node are a common scenario in our clinical practice. Hence, the geometry of the actual clinical cases is likely to fall within the range of the constituent plan library of the model. While most of the authors have developed models specific to left-sided breast cancer, including the current one, others have developed a generalized model. Foglia et al. has developed model for patients with breast conservation requiring tumor bed boost and suggested to conduct dedicated studies for other settings (14). Though few studies have developed models for RNI, most of them are either in the setting of breast conservation or has been done using conventional fractionation in non-Varian treatment planning system viz., the MD Anderson Cancer Center Auto Plan (MDAP) system and the Pinnacle treatment planning system (TPS) (21). Hence the study methodology is not comparable with the RapidPlan™ based modelling presented here. Moreover, only 20 patients were used to develop the model though it was validated in 10 right and 10 left chest wall patients. Fewer dosimetric parameters were used for the comparative analysis. Similar to our study, they also reported that quality plans can be generated by AI-based automatic planning systems with clinical efficiency. However, in view of the above shortcomings, the results of the current study are more robust compared to the MD Anderson report.

**Table 3:** Review of studies published on rapid planning for breast cancer.

Author	Year	N for model/validation	Laterality	Target volumes	Guidelines	Metrices compared	Dose prescription/technique	External validation	KBP performance	Time reduction
Fogliata (14)	2015	150/50	Both and bilateral	Breast and boost	In-house	18	40.5 in 15 for breast and 48 in 15 for boost, VMAT in Eclipse v 13.5	Yes	7 no difference, 11 better in KBP	Not reported
Wang (15)	2017	80/10	Left	Breast and boost	In-house	16	45 in 25 for breast and 60 in 25 for boost, VMAT in Eclipse v 13.5	No	No difference for senior planners	Not reported
Ben Archibald-Heeren (16)	2020	100/0	Both	Breast alone	RTOG/ESTRO	39	50 in 25 and 42.4 in 16, Hybrid IMRT tangent RT in Raystation	No	23 no difference, 14 better in KBP and 2 better in CP	23 to 5 mins
Inoue (17)	2020	20/5	Left and bilateral	Breast alone	RTOG	9	50 in 25, VMAT in Eclipse v 15.6	No	7 no difference, 2 better in KBP	120 to 15 mins
Costa (18)	2021	56/20 each for Truebeam and Halcyon	Left	Breast/SCF/AX/IMN	ESTRO	28	40 in 15 or 50 in 25 or 50.4 in 28 for breast with 63 in 28 for boost, VMAT in Eclipse v 15.6	No	Comparison with CP not shown. Truebeam and Halcyon plans compared	Not reported
Rago (19)	2021	52-120/40	Both	Breast/Boost/SCF/IMN	In-house	32	40 in 15 or 50 in 25 for breast with 57.5-62.5 in 25 for boost, VMAT in Eclipse v 15.6	Yes	24 no difference, 8 better in KBP	Not reported
Apaza Blanco OA (20)	2021	50/20	Both	Breast alone	AAPM report TG-263	8	three dose levels in 20 fractions. (CTV_SIB) dose prescription of 56Gy, proximal CTV of 46 Gy and (CTV_ distal) of 43 Gy. Ecclipse,VMAT	No	6 out of 8 are better Increase in ipsilateral lung dose and contra breast for	30% for beginner
Jiang (21)	2022	20/20	both	Chest wall/SCF/IMN	ESTRO	10	50 in 25, MD Anderson Cancer CenterAutoPlan (MDAP) system and Pinnacle treatment planning system (TPS), (v9.8, Philips Radiation Oncology Systems, Fitchburg, WI)	NO	Same or better in 5,	reported
Current	2021	133/23	Left	Chest wall/SCF/IMN	ESTRO	20	40 in 15 or 26 in 5, VMAT in Eclipse v 16.1	No	same or better in 15, CP better in 4	4 to 5 hrs to 20 minutes

To improve the efficiency of breast treatment planning using KBP for VMAT, it is crucial to standardise various essential steps involved in the process.

1) First and foremost is the selection of cases for the model training: Minimum number of patients required for configuring KBP models is 20, however adding more plans usually helps in creating a more robust plan (13). We have taken 60 and 73 patients selected for generating the KBP models for 40 Gy in 15 fractions and 26 Gy in 5 fractions model respectively.The robustness and accuracy depend on the quality of plans used for model training and configuration. Wang et al. has also stated that suboptimal plans when used in model configuration can degrade the KBP predicted plans. He also emphasized on the requirement of deeper analyses on the goodness of the estimation model configuration in terms of the model size, plan and anatomy homogeneity (15).
2) Uniform adoption of nomenclature for target volumes and OAR: This is a very basic requirement when selecting patients for model training. It reduces errors and also forms the basis for future validation of the model for internal and external use. Adoption of uniform nomenclature (codes) for target volumes and OARs helps in clinical workflow by automatic structure matching in DVH estimation models. To take full advantage of automatic structure matching, define a code for each plan structure of the structure template, such that all plan structures will have the same structures codes as the ones introduced in the model. If a plan structure has no code, matching is based on the structure identifier. Template structure code assignment is recommended for robust structure matching between the new cases and model structures.
3) Choice of the proper objectives and priorities: The judicious use of optimization objectives while creating a model is considered crucial for model quality. Many authors have observed that OAR doses from the CLI plans and KBP plans (plans generated using line constraints and auto-generated priorities of the KBP model) were similar (22, 23). Rago et al. have also reported that the good results of the plans generated with KBP could come from the combination of the two objectives included in the model: the generated line-objective and the mean objective, both with generated priorities (19). As difference in prescription dose and number of fractions is very large in 40Gy/15 Fr and 26Gy/5Fr, difference in mean dose to the OARs are also quite large, so we have not made a generalized model. In this study, line objectives and mean objectives with generated priorities have been used for OARs.
4) Standardization of other planning parameters: Treatment planning parameters like the number of patients, beam energy, number of arcs, etc. are directly linked to the performance of the KBP models and their prediction ability. In this study, we utilized the optimal number of clinically approved treatment plans and used a flattened 6 MV beam for VMAT plans with 2 to 4 partial arcs.

Predicted gains from model-based planning and their impact on clinical workflow: Overall, plans generated by both the models (Model 1 and Model 2) are considered clinically acceptable based on the clinical goals and comparable dose distribution. Except for the increase in the dose of the ipsilateral lung which is less than 1 Gy (**table 2**), most of the dosimetric parameters were either comparable or better with the KBP plans. Blanco, et al. also reported significant increase in dose of ipsilateral lung in the KBP plans favoring plans of manual plan in their study (24). Many authors accepted skilled manual interventions on the KBP plans to achieve high quality results. When there is very close proximity or overlap of OARs with PTVs, minor refinements can be considered to support clinical decisions to compromise either coverage or OAR constraints (18, 19).

So Ipsilateral lung dose can be reduced by replanning KBP plans. Swamidas et al. also suggested that dose to a particular OAR can be reduced by replanning KBP plans where the optimization objectives/priorities were manually tweaked such that the DVH of the OAR to be at the lower border of the estimation band of DVH prediction without compromising the target coverage, with a single optimization (25). Hence, these models can be considered acceptable for clinical implementation. Model-generated plans are also likely to improve the workflow by giving consistent plans, especially in this group of post-mastectomy patients wherein complex treatment volumes are very common. In the CLI plans, many optimization structures were created to control the dose spill to the heart and lungs, and it is explained in the literature (4). However, for KBP plans, the creation of such optimization structures is not necessary with RapidPlan™ as predicted DVH objectives automatically take into account the dose spill and control it accordingly (26). It also directly helps in reducing the planning time. The time taken for plan generation has shown a considerable reduction, from 4-5 hours to 20-30 minutes. The quality of plans generated using the model is independent of the planner. Another important gain reported in our study is the generation of treatment plans with reduced monitor units and less complexity, thereby further improving the treatment planning and delivery efficiency. Many authors reported findings similar to our results. Tamura et al. have reported that two full arcs VMAT plans generated by the KBP might decrease the MU and the modulation complexity (24). In our case also, all the plans are generated with two to four partial arcs. On the other hand, Hussein et al. also showed the MU and modulation complexity were not different between KBP and clinical plan for two full arcs VMAT plans. In his study, KBP plans for prostate cancer are with lesser MU while that for cervix,KBP plans are with higher MU compared to manual plan (26). Kubo et al. also have stated that RapidPlan™ might reduce plan complexity when appropriate objective constraints are used (27). which corroborates with our experience. Swamidas et al, also reported our institutional experience on development and validation of KBP for cervical cancer and similarly found significantly lesser monitor units in KBP plans as compared to manual plans (25).

The blinded review by the consultants helped in removing any biases towards any planning technique. The results obtained showed that both the CLI and KBP plans are clinically acceptable. The sparing of ipsilateral lung was found to be lower with KBP. However, in absolute terms, the difference was less than 1 Gy. Though it can be argued that the risk of toxicity, especially radiation induced pneumonitis, will be higher, the clinical manifestation is very rare. The incidence of pneumonitis is low at our institute and the safety of IMRT for treatment of breast cancer has been reported earlier by us (28).

## Limitation

As this model was made for a selected cohort of patients, its applicability to right-sided disease, breast conservation, bilateral disease, and prescriptions with multiple dose levels has not been tested. Moreover, prospective internal and external validation of the model is strongly recommended for a larger number of patients. It would also be worthwhile to quantify the time saved by employing KBP planning in clinics.

## Conclusion

Knowledge based planning models for VMAT technique for dose prescriptions of 26 Gy in 5 fractions and 40 Gy in 15 fractions have been developed and validated for breast cancer involving the left chest wall, internal mammary chain and supraclavicular fossa. This has the potential to improve the work flow for VMAT planning involving moderately hypo fractionated and ultra-hypo fractionated radiotherapy regimens.

The model generated plans were comparable with the clinical plans generated by experienced physicists in terms of dose distribution. The KBP plans were found to be less complex and passed the deliverability quality assurance tests and, hence can be clinically implemented.

## Data availability statement

The original contributions presented in the study are included in the article/**Supplementary Material**. Further inquiries can be directed to the corresponding authors.

## Author contributions

All authors listed have made a substantial, direct, and intellectual contribution to the work and approved it for publication.
