# Supplementary material for: Can knowledge based treatment planning of VMAT for post-mastectomy locoregional radiotherapy involving internal mammary chain and supraclavicular fossa improve performance efficiency?
Source: Front Oncol. 2023 Apr 3;13:991952. doi: 10.3389/fonc.2023.991952 (PMC10128860; doi:10.3389/fonc.2023.991952)
Supplement: Supplementary file 1 [file DataSheet_1.docx]

**Supplementary Tables**

Supplementary table 1:

|  | LEFT CW_26Gy/5# | | | LEFT CW_40Gy/15# | | |
| --- | --- | --- | --- | --- | --- | --- |
| **OAR's** | **R^2^** | **χ^2^** | **MSE** | **R^2^** | **χ^2^** | **MSE** |
| HEART | 0.527 | 1.065 | 0.03 | 0.539 | 1.063 | 0.02 |
| CONTRA LUNG (RT) | 0.758 | 1.047 | 0.02 | 0.536 | 1.037 | 0.01 |
| IPSILATERAL LUNG (LT) | 0.399 | 1.062 | 0.06 | 0.535 | 1.107 | 0.06 |
| CONTRA BREAST | 0.217 | 1.023 | 0.01 | 0.342 | 1.05 | 0.01 |

Supplementary Fig1a: Average DVH comparisons for different OARS and PTVs (40Gy15fr)


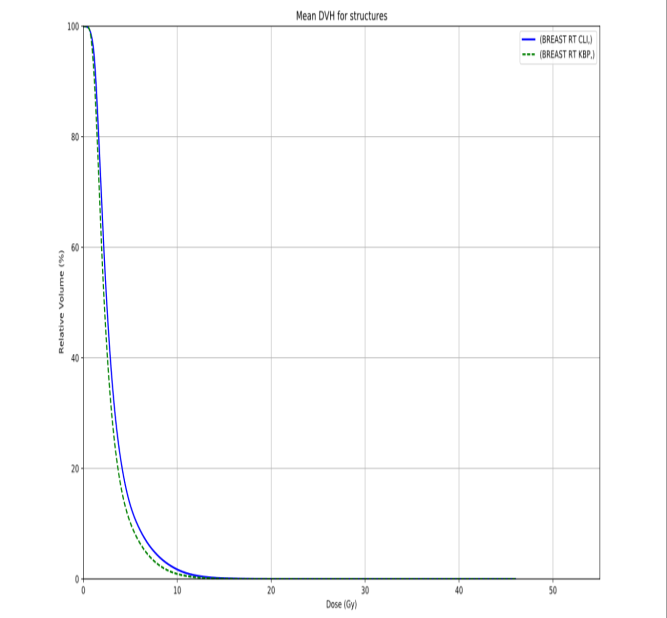

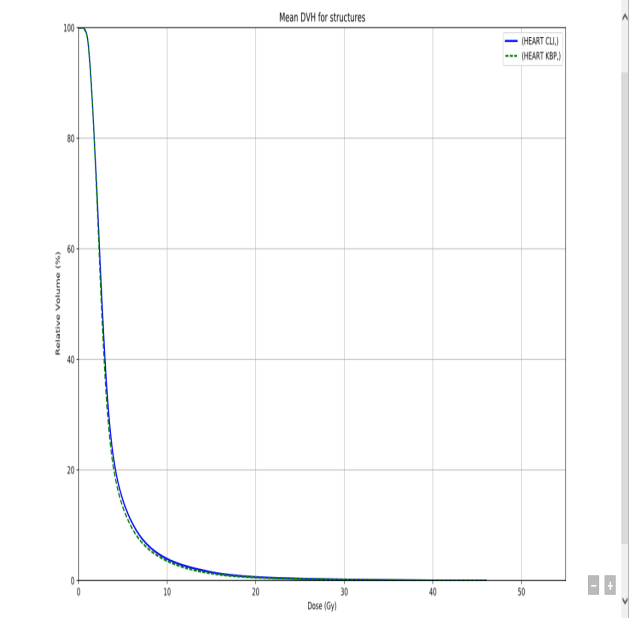


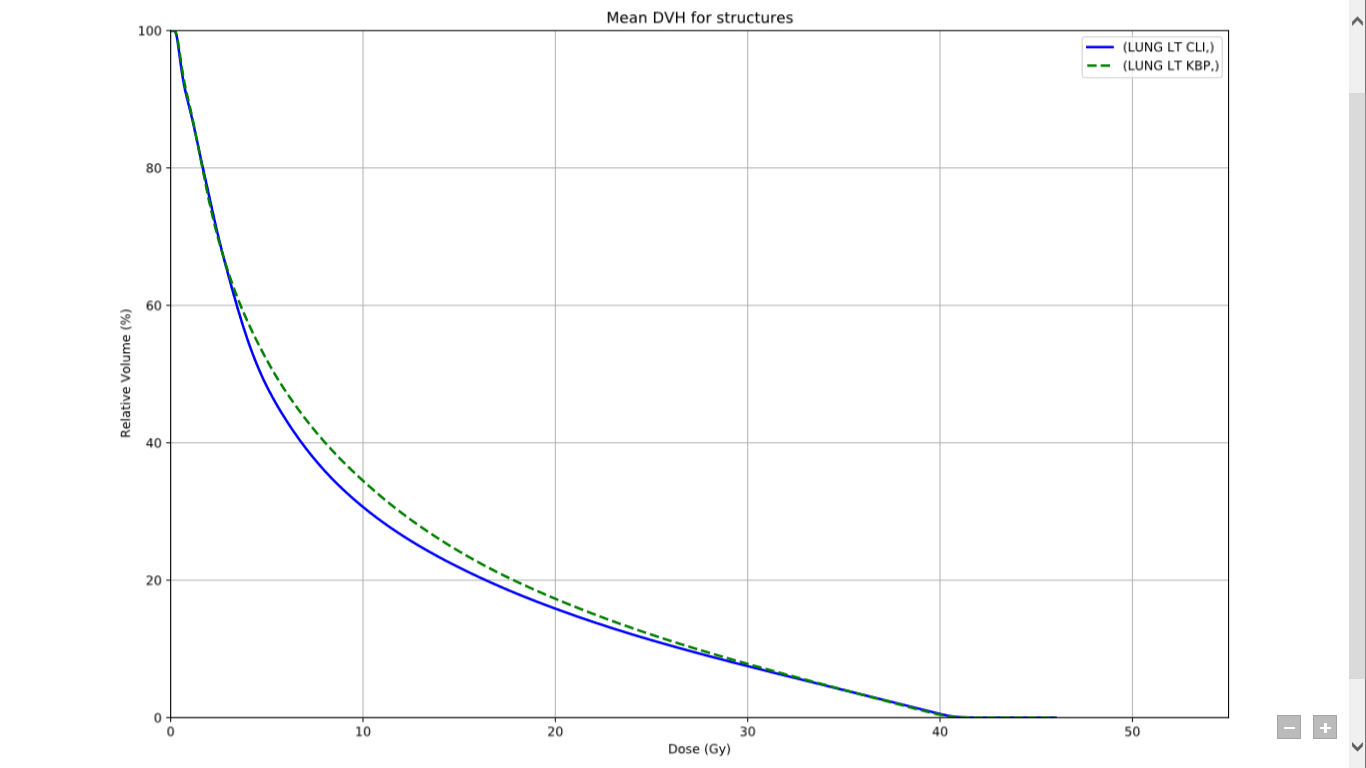

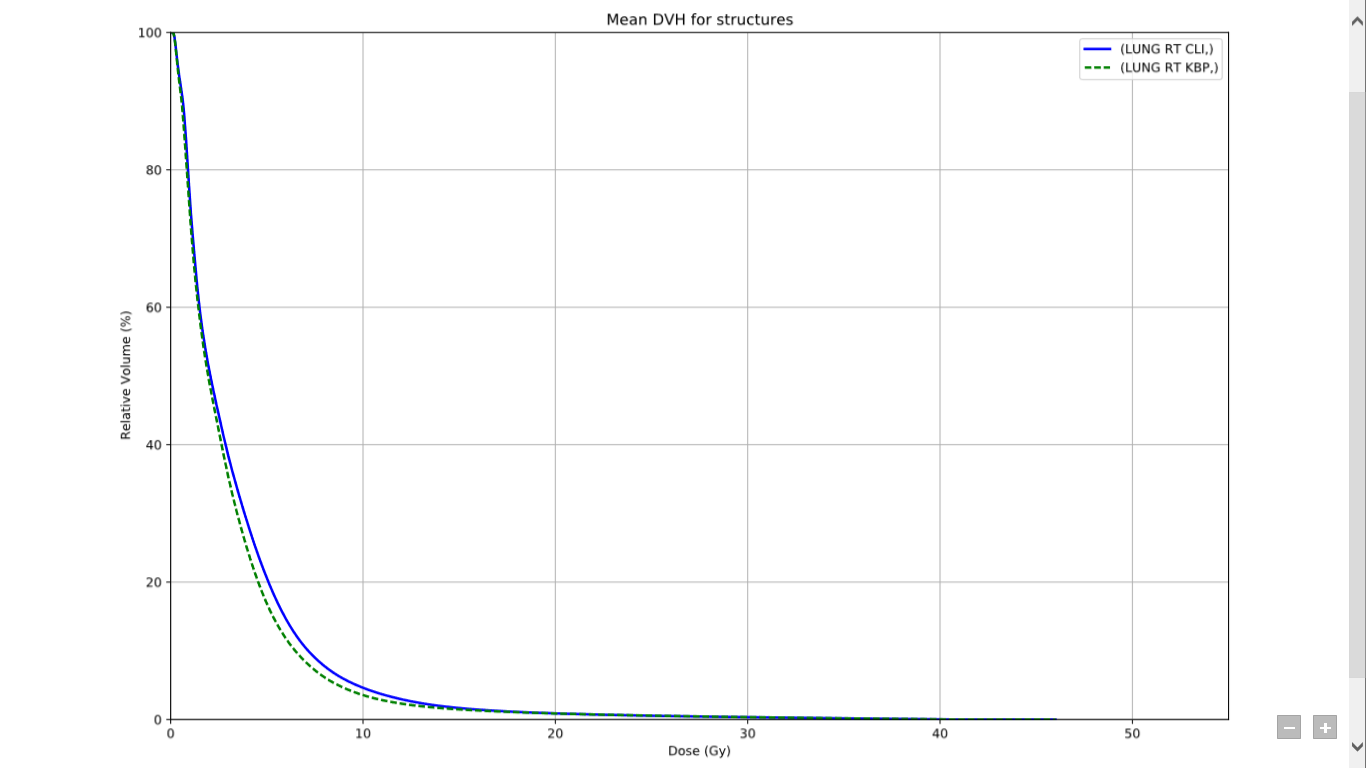


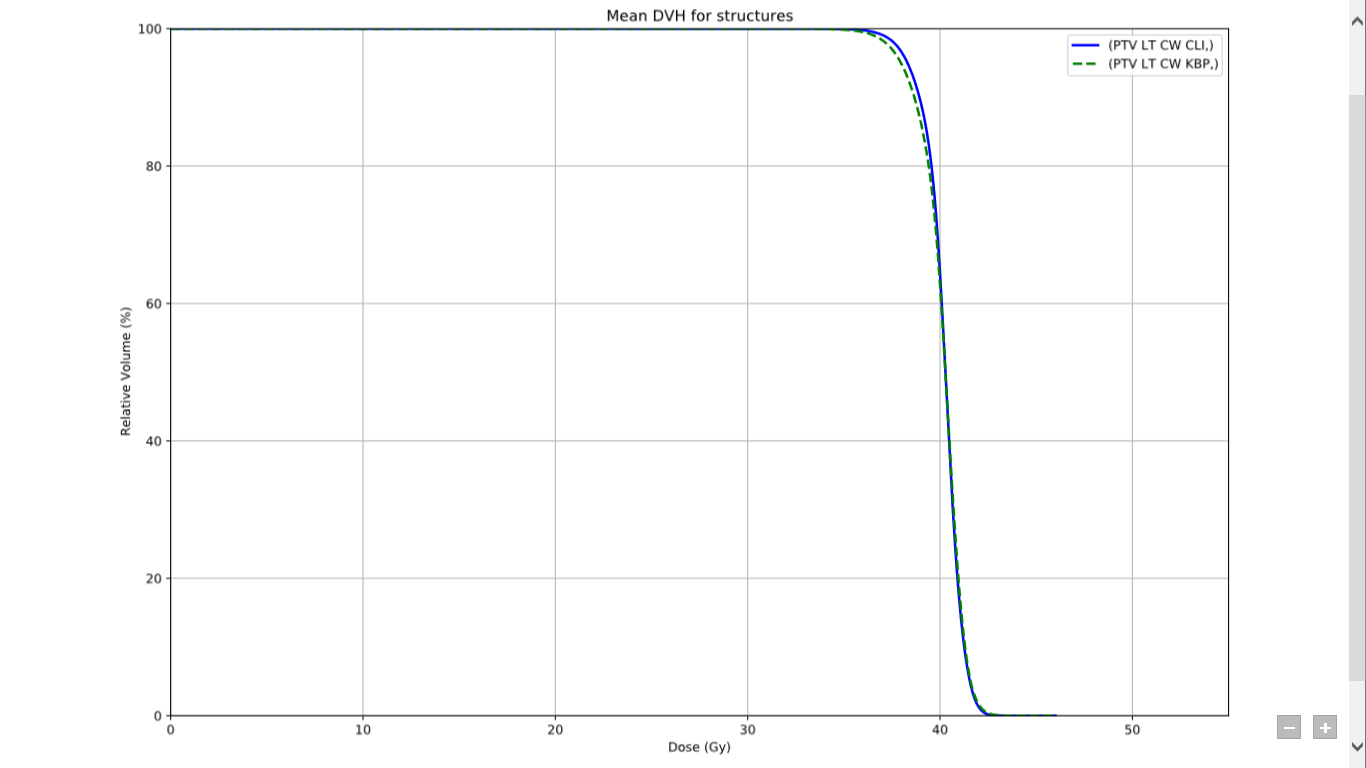

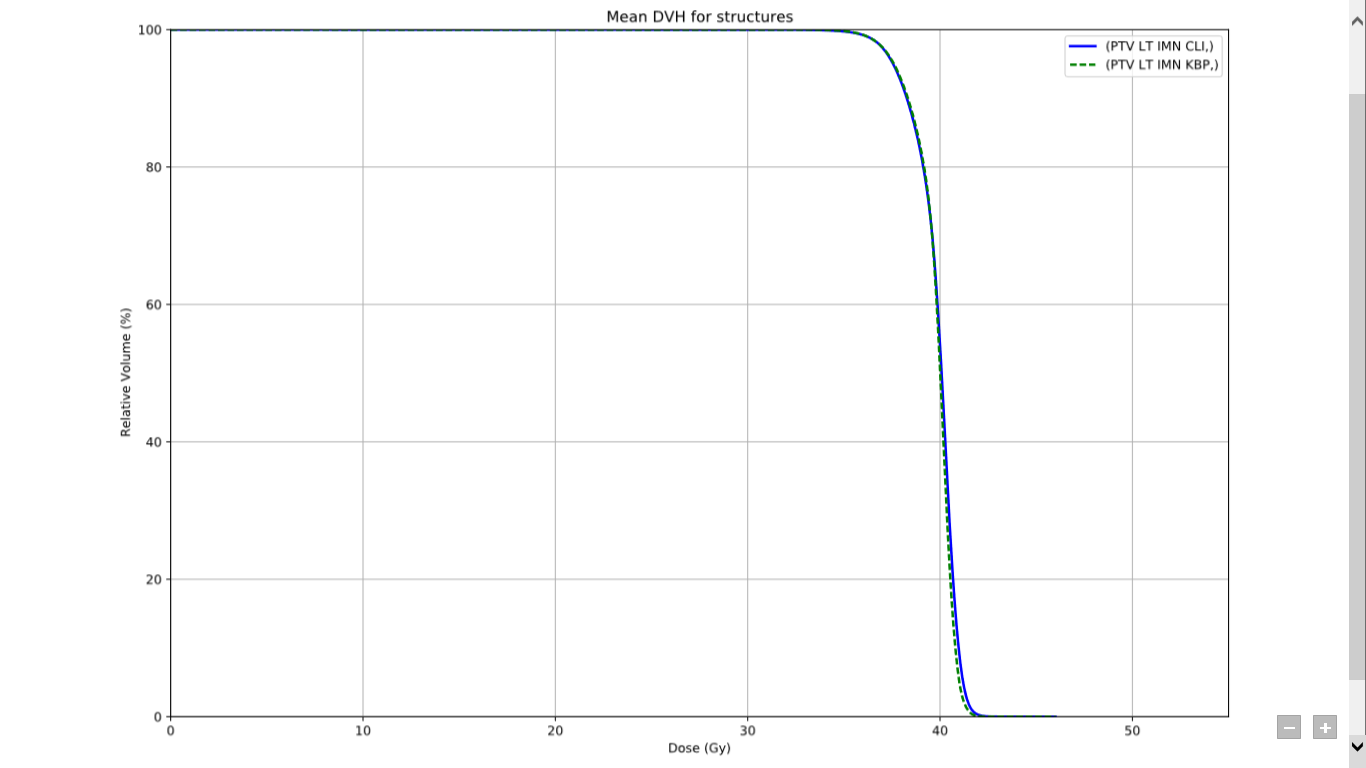

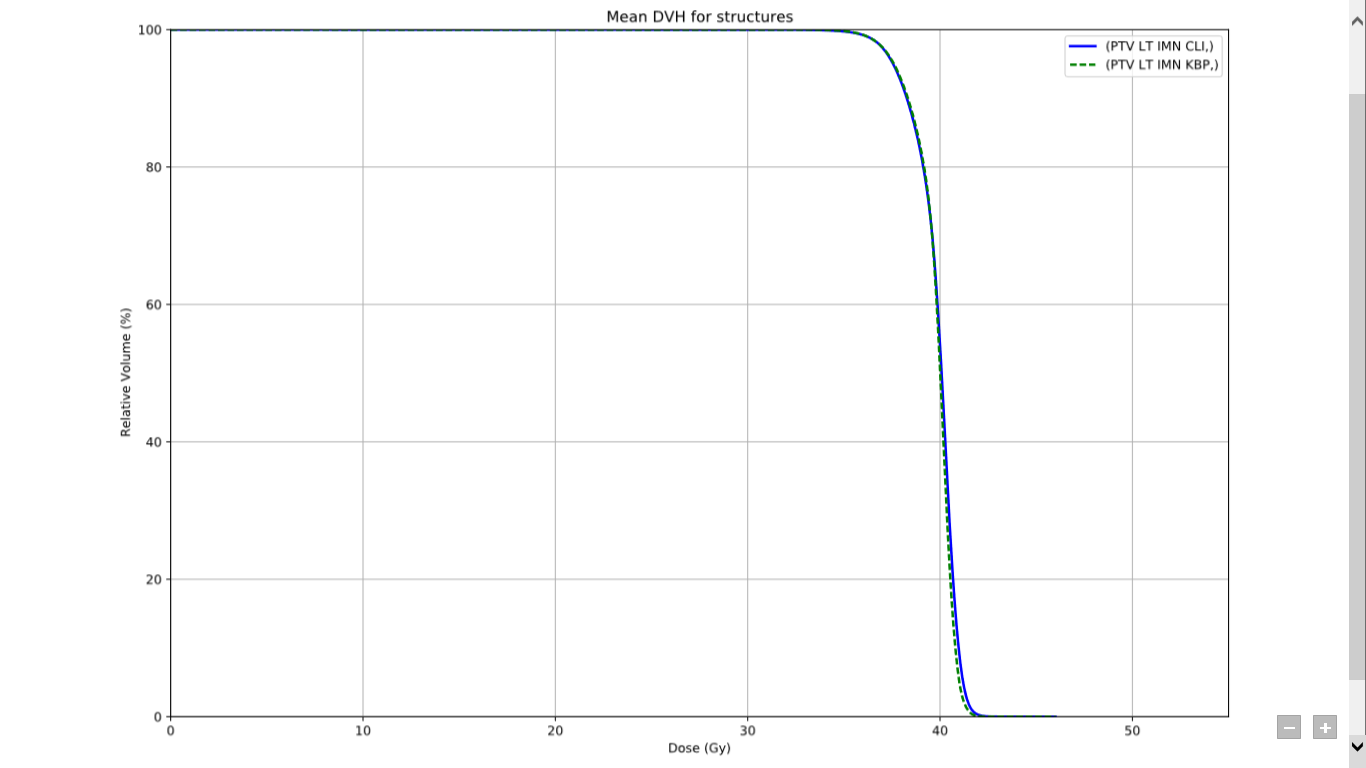

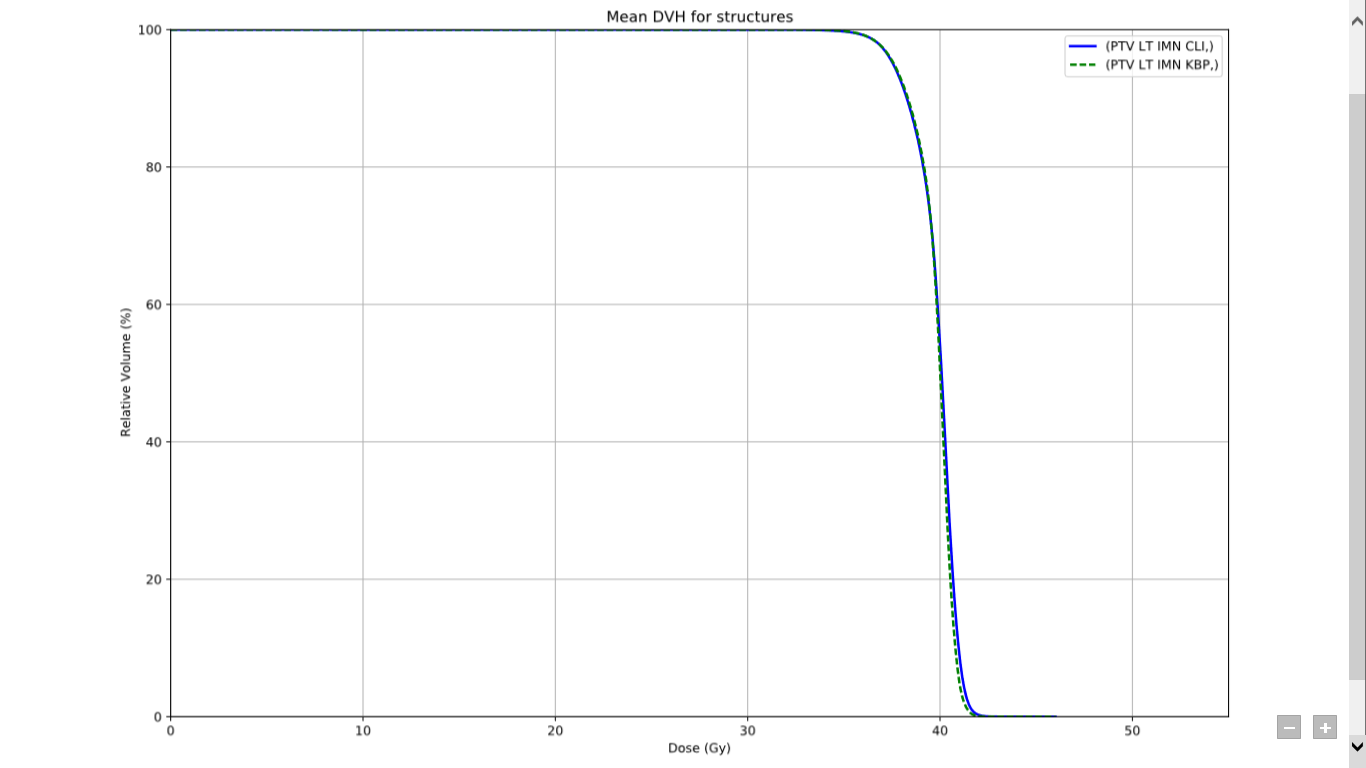


Supplementary Fig1b: Average DVH comparisons for different OARS and PTVs (

26Gy#5fr)


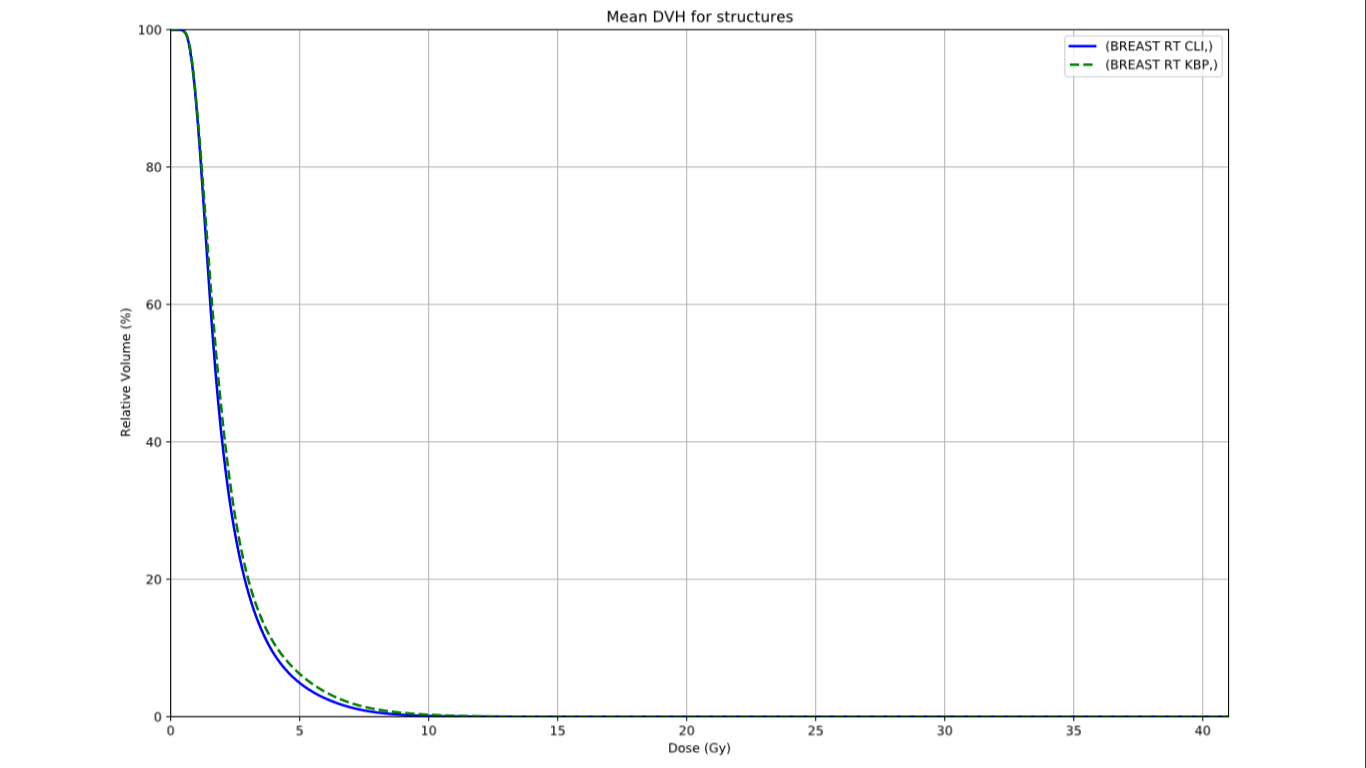

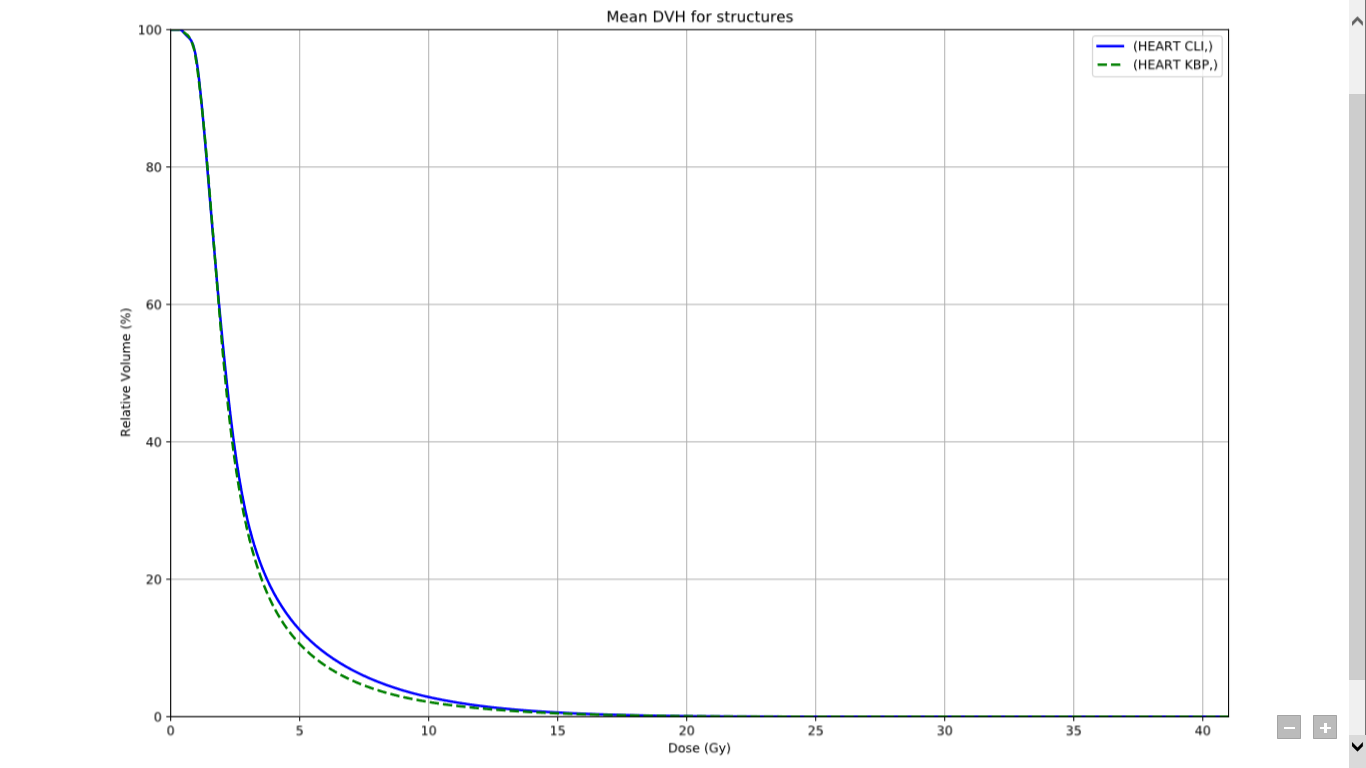

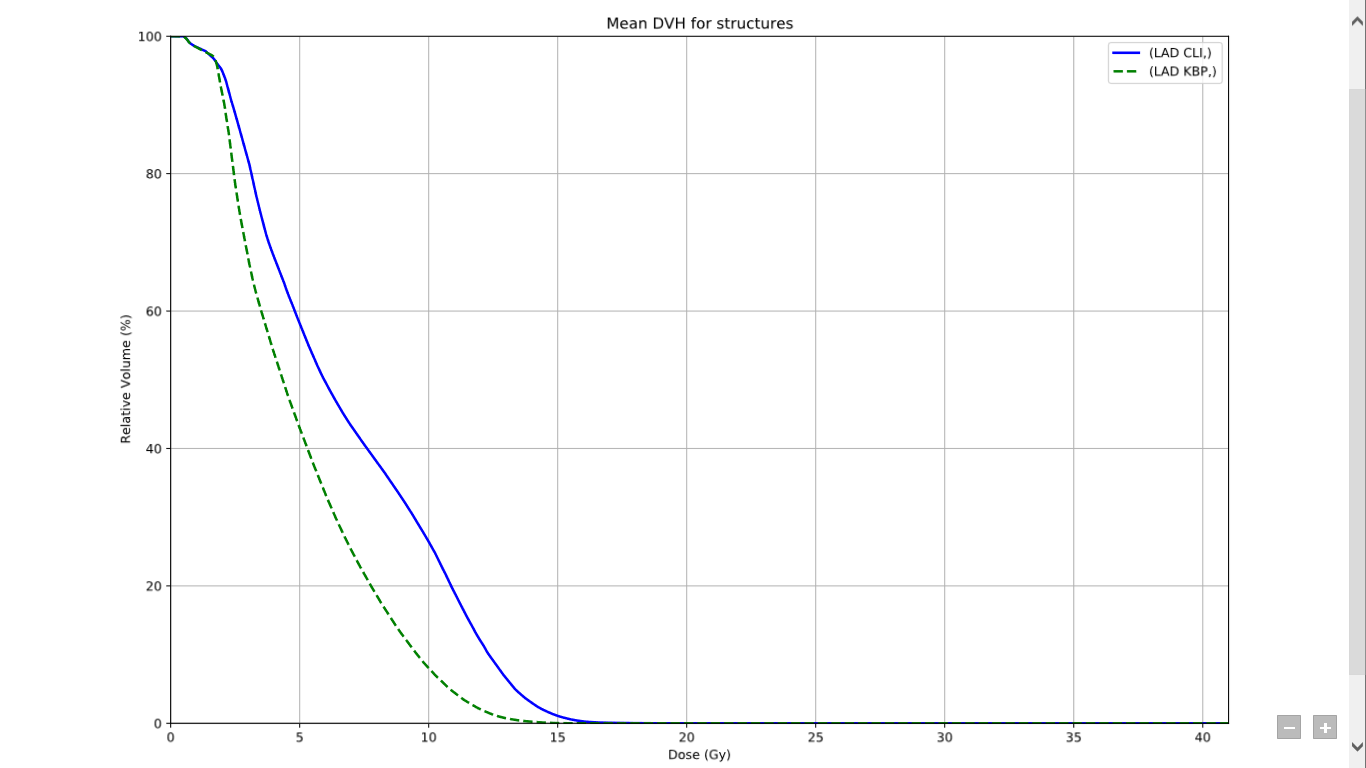

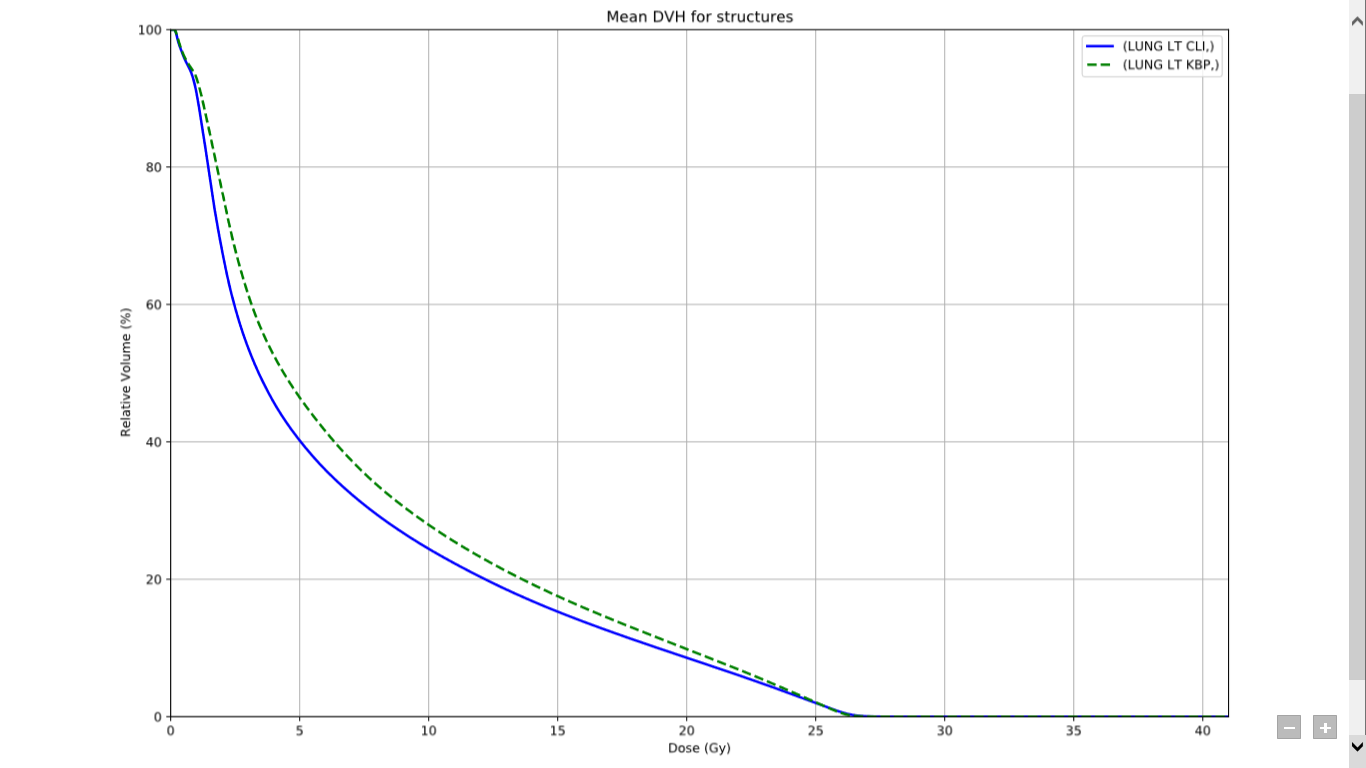

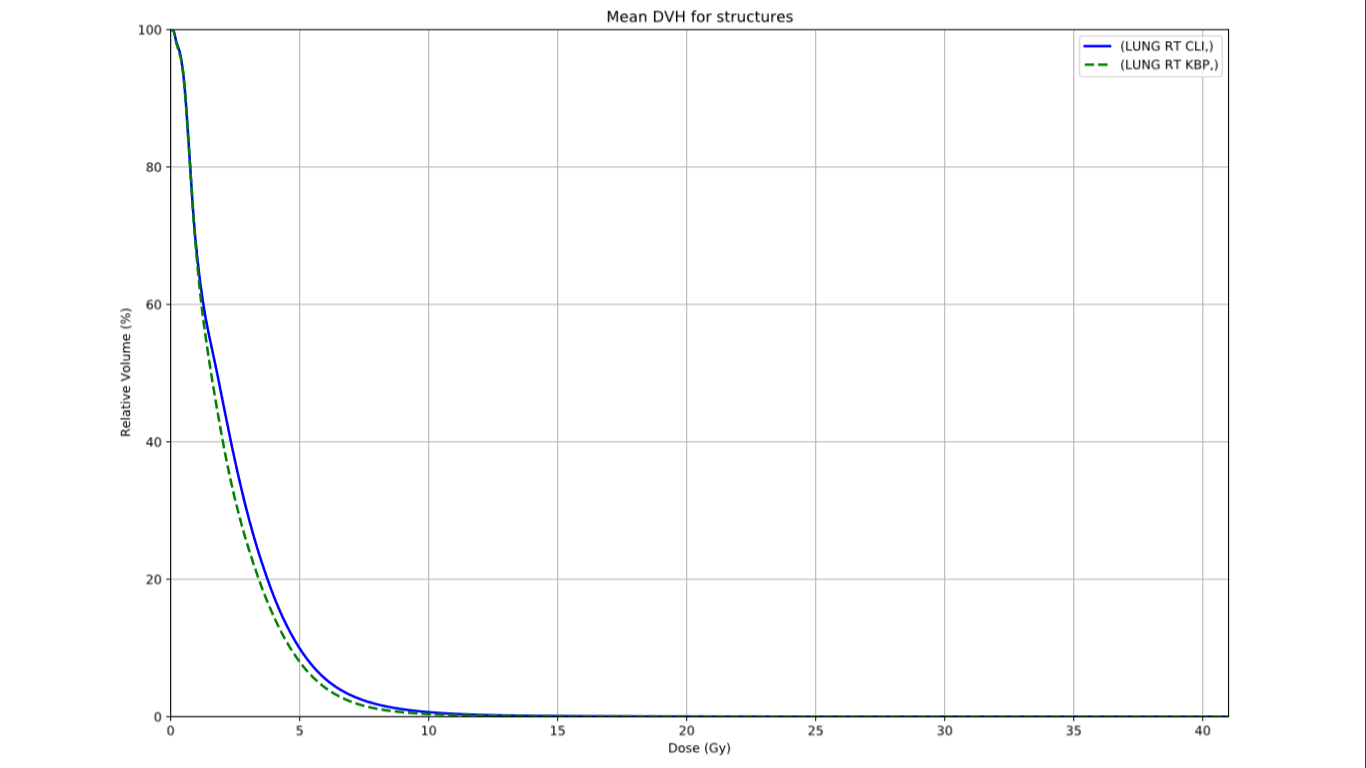

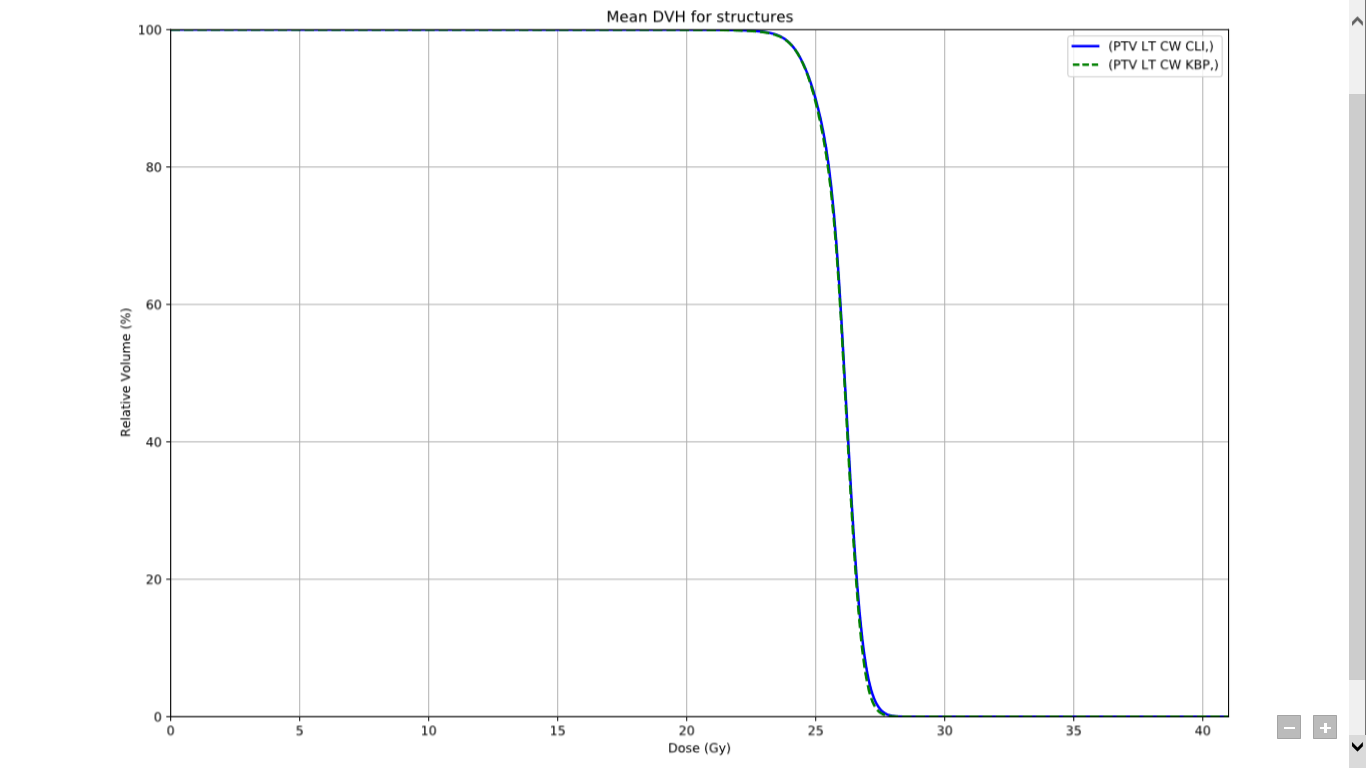


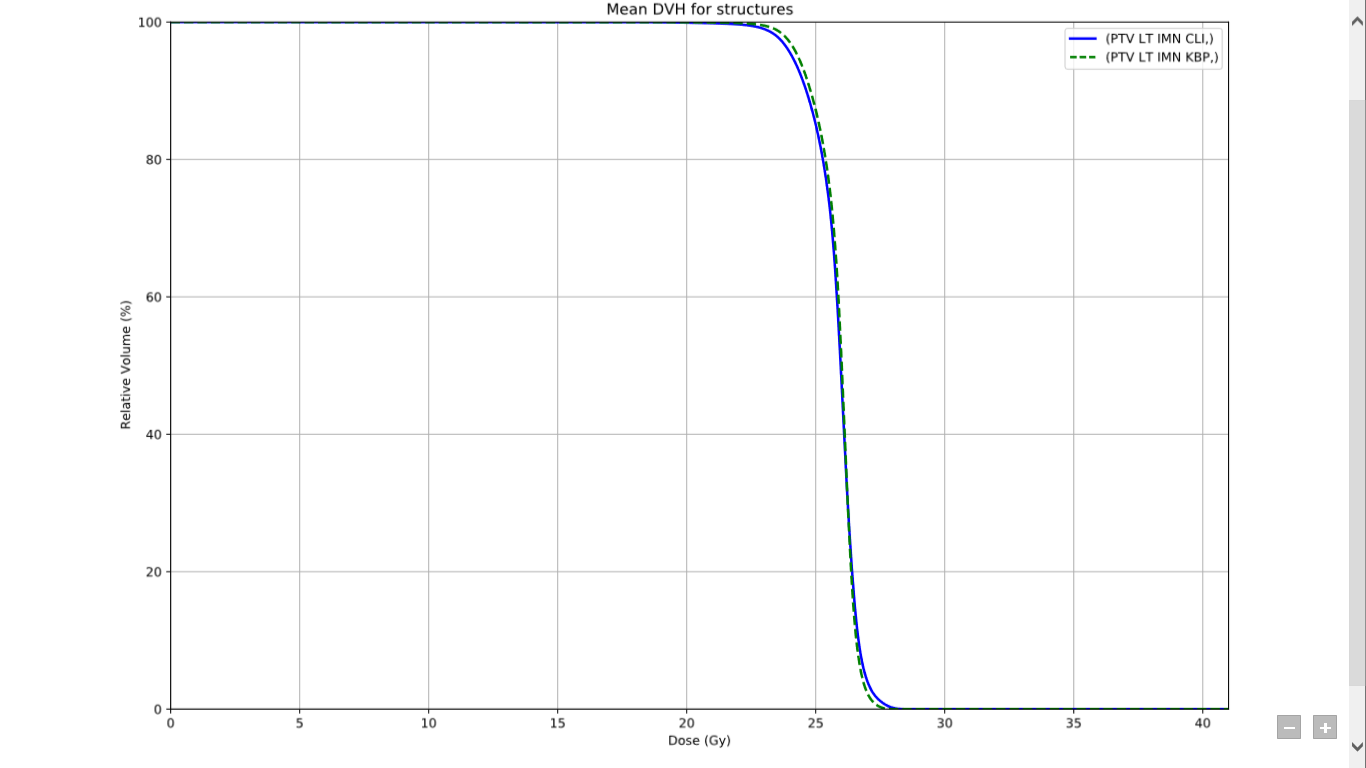


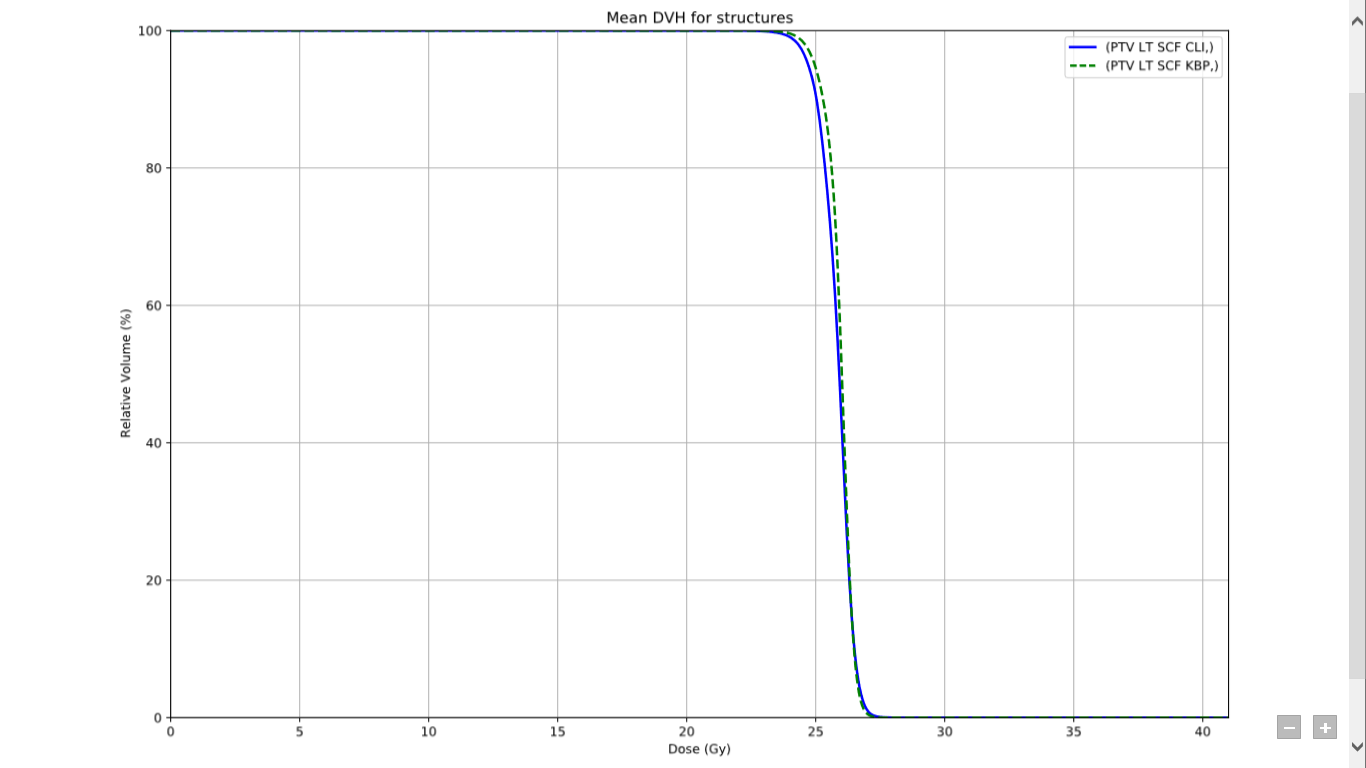


Supplementary table 2a MU AND COMPLEXITY/DQA analysis 26 Gy#5 Fr

| Patien1 | Complexity  metric_CLINICAL | Complexity  Metric_KBP | MU_Cli | MU_KBP | AD_Gamma_clinic | AD_gamma_KBP |
| --- | --- | --- | --- | --- | --- | --- |
| 1.00 | 0.16 | 0.11 | 2196.14 | 1621.59 | 99.30 | 98.30 |
| 2.00 | 0.15 | 0.12 | 1985.87 | 1654.40 | 98.10 | 98.40 |
| 3.00 | 0.14 | 0.16 | 2511.28 | 2687.69 | 98.80 | 99.00 |
| 4.00 | 0.13 | 0.13 | 1693.99 | 1574.37 | 98.30 | 97.60 |
| 5.00 | 0.13 | 0.12 | 1829.41 | 1862.17 | 99.00 | 98.10 |
| 6.00 | 0.16 | 0.14 | 2206.56 | 1743.10 | 98.40 | 98.90 |
| 7.00 | 0.15 | 0.11 | 2342.65 | 1635.72 | 98.10 | 98.70 |
| 8.00 | 0.12 | 0.11 | 1773.71 | 1587.01 | 99.80 | 98.50 |
| 9.00 | 0.14 | 0.12 | 1623.86 | 1527.75 | 99.30 | 99.80 |
| 10.00 | 0.16 | 0.12 | 1975.70 | 1507.84 | 99.10 | 99.40 |
| 11.00 | 0.10 | 0.12 | 1285.01 | 1485.55 | 100.00 | 99.60 |
| 12.00 | 0.15 | 0.11 | 2046.83 | 1514.16 | 99.50 | 99.30 |
| 13.00 | 0.17 | 0.14 | 2093.04 | 1757.06 | 98.80 | 99.10 |
| Avg | 0.14 | 0.12 | 1966.47 | 1704.49 | 98.96 | 98.82 |
| Std dev | 0.02 | 0.01 | 327.06 | 315.53 | 0.62 | 0.63 |

Supplementary table 2b MU AND COMPLEXITY/DQA analysis 40 Gy#15 Fr

| Patien1 | Complexity  metric_CLINICAL | Complexity  Metric_KBP | MU_Cli | MU_KBP | AD_Gamma_clinic | AD_gamma_KBP |
| --- | --- | --- | --- | --- | --- | --- |
| 1.00 | 0.13 | 0.12 | 1044.34 | 949.49 | 98.00 | 99.60 |
| 2.00 | 0.17 | 0.13 | 900.18 | 789.59 | 96.90 | 96.90 |
| 3.00 | 0.14 | 0.14 | 1314.36 | 1301.36 | 99.30 | 99.50 |
| 4.00 | 0.12 | 0.11 | 854.08 | 799.50 | 98.90 | 99.30 |
| 5.00 | 0.14 | 0.13 | 890.94 | 821.30 | 95.10 | 96.60 |
| 6.00 | 0.12 | 0.13 | 855.05 | 902.65 | 96.70 | 97.10 |
| 7.00 | 0.14 | 0.11 | 965.75 | 874.49 | 98.00 | 98.40 |
| 8.00 | 0.11 | 0.11 | 873.11 | 832.40 | 98.90 | 98.90 |
| 9.00 | 0.13 | 0.12 | 921.17 | 845.59 | 99.70 | 99.60 |
| 10.00 | 0.13 | 0.12 | 962.37 | 862.33 | 97.70 | 97.70 |
| Avg | 0.13 | 0.12 | 958.14 | 897.87 | 97.92 | 98.36 |
| Std dev | 0.02 | 0.01 | 138.37 | 149.66 | 1.40 | 1.19 |

| **LEFT CW 26GY/5F** | | | | | |
| --- | --- | --- | --- | --- | --- |
| **Structure** | **Objectives** | **Vol(%)** | **Dose** | **Priority** | **gEUD a** |
| PTV LT CW | Upper | 0 | 26 | 100 |  |
|  | Lower | 100 | 26 | 100 |  |
| PTV LT IMN | Upper | 0 | 26 | 65 |  |
|  | Lower | 100 | 26 | 65 |  |
| PTV LT SCF | Upper | 0 | 26 | 80 |  |
|  | Lower | 100 | 26 | 80 |  |
| Breast Rt | Mean |  | 2 | 62 |  |
|  | Upper gEUD |  | 2 | 62 | 1 |
|  | Line (preferring target) | Generated | Generated | 62 |  |
| Heart | Mean |  | 2.665 | 62 |  |
|  | Upper gEUD |  | 2.665 | 62 | 1 |
|  | Line (preferring OAR) | Generated | Generated | 62 |  |
| LAD | Upper | 0 | 15.6 | 62 |  |
|  | Upper gEUD |  | 12 | 62 | 40 |
|  | Line (preferring target) | Generated | Generated | 62 |  |
| Liver | Line (preferring target) | Generated | Generated | 25 |  |
| Lung Lt | Upper | 55 | 3 | 60 |  |
|  | Upper | 35 | 6 | 60 |  |
|  | Upper | 17 | 12 | 62 |  |
|  | Mean |  | 7 | 60 |  |
| Lung Rt | Mean | 2 | 62 |  |  |
|  | Upper gEUD | 2 | 62 | 1 |  |
|  | Line (preferring target) | Generated | Generated | 62 |  |
| Spinal Cord | Upper | 0 | 12 | 50 |  |
|  | Line (preferring target) | Generated | Generated | 50 |  |
| Thyroid | Upper | 0 | 26 | 50 |  |
| Esophagus | Upper | 0 | 26 | 50 |  |

Supplementary table 3: Dose volume objectives and priorities used in knowledge based planning,

| **LEFT CW 40GY/15F** | | | | | |
| --- | --- | --- | --- | --- | --- |
| **Structure** | **Objectives** | **Vol(%)** | **Dose** | **Priority** | **gEUD a** |
| PTV LT CW | Upper | 0 | 40.2 | 105 |  |
|  | Lower | 100 | 40.1 | 110 |  |
| PTV LT IMN | Upper | 0 | 40 | 75 |  |
|  | Lower | 100 | 40 | 80 |  |
| PTV LT SCF | Upper | 0 | 40 | 80 |  |
|  | Lower | 100 | 40 | 85 |  |
| Breast Rt | Mean |  | 3 | 60 |  |
|  | Upper gEUD |  | 2.75 | 62 | 1 |
|  | Line (preferring target) | Generated | Generated | 62 |  |
| Heart | Mean |  | 3 | 60 |  |
|  | Upper gEUD |  | 2.9 | 62 | 1 |
|  | Line (preferring OAR) | Generated | Generated | Generated |  |
| LAD | Upper | 0 | 17 | 65 |  |
|  | Upper gEUD |  | 16 | 65 | 40 |
|  | Line (preferring target) | Generated | Generated | 62 |  |
| Liver | Line (preferring target) | Generated | Generated | Generated |  |
| Lung Lt | Mean | 55 | 3 | 60 |  |
|  | Upper gEUD | 35 | 6 | 60 |  |
|  | Line (preferring target) | Generated | Generated | Generated |  |
| Lung Rt | Mean |  | 4 | 60 |  |
|  | Upper gEUD |  | 3.8 | 62 | 1 |
|  | Line (preferring target) | Generated | Generated | Generated |  |
| Esophagus | Upper | 0 | 39 | 50 |  |
| Spinal Cord | Upper | 0 | 20 | 50 |  |
|  | Line (preferring target) | Generated | Generated | Generated |  |
| Thyroid | Upper | 0 | 39 | 50 |  |

**Appendix 1**

**Basic Principle of Knowledge based planning module used in the current study**

**( Rapid plan, Varian Medical Systems)**

Rapid plan is a DVH estimation model which is used to predict DVHs for treatment plans. The model is configured based on the previous experience from similar treatment plans called training plans. The training plans are selected according to the purpose of the model, which defines whether the model will be used for a wide variety of patients with different organ sizes, field geometry and dose prescriptions, or for more specific cases. The training plans, should be clinically acceptable and meet the clinical goals.

The Rapid Plan engine consists of three major components: i) a module for model training, that deals with the data modelling to train and predict the DVH for each OAR ii) a module for estimation of the DVH based on the predictive model and iii) Generation of dose-volume constraints to take into account the individual patient geometry for plan optimization and obtaining the actual DVH.

For model configuration, information related to each structure set, dose, and field geometry were extracted and are converted into characteristic curves and parameter values. For each training plan and for each structure matched to any model OAR, the data extraction phase divides the volume of the structure into functionally different regions such as, Out of fled region, Leaf-transmission region, In field region and overlap region. For each region, the relative volume and cumulative volume histogram of the given dose matrix is calculated. Similarly, the geometric distribution of the region is evaluated as a cumulative volume histogram of Geometry –Based Expected Dose (GED) matrix. The GED metric represents how far away the voxels in the structure are from the target’s surfaces. The “distance” value consists of the amount of dose that each target contributes to a voxel given the current field geometry. The metric depends solely on the patient anatomy; the desired dose to deliver to every target (target level) and the field setup (position and orientation). The GED metric resembles a conformal dose.

The model training phase produces a DVH estimation model, which uses a combination of Principal Component Analysis (PCA) and regression techniques. The model training phase applies the PCA to the GEDs of all OARs to extract the information (features) contained in the GEDs. The same procedure is applied to the DVHs. The extracted principal component scores of the GED are then combined with the anatomical features of the patient's OAR and the target volume voxels, followed by the model training phase that applies stepwise regression for each principal component of the DVH. Finally, the stepwise regression model is constructed using an iterative process of forward and backward methods until convergence.

The DVH estimation component of the algorithm is used for generating estimated DVHs and optimization objectives for a plan, which consists of the estimation generation phase, and the objective generation phase. The estimation generation phase calculates for each structure the same metrics that were calculated during the data extraction of the DVH estimation model.

Rapidplan offers the capability for the user to analyse the model quality, where possible outlier plans are identified and managed. The model quality is given by a summary of goodness-of-fit and goodness of estimation statistics. These include, among others, per each trained OAR, the coefficient of determination R^2^ (goodness-of-fit), and the mean squared error MSE between the original and estimate (goodness-of-estimation). The R^2^ describes how well the regression model represents the training plan data, by quantifying the variability in the data expressed by the model; its value ranges from 0 to 1: larger R^2^ indicates a better model fit, however, R^2^ values too close to 1 could be a symptom of overfitting. The MSE describes how well the model is able to estimate the original DVH in a training plan, by measuring the distance between the original DVH and the mean of the upper and lower bounds of the estimated DVH; the closer the value is to 0, the better is the estimation capability of the model.
